# T‐cell differentiation stage block bias confers hypermethylation and mediastinal preference in T‐cell lymphoblastic lymphoma

**DOI:** 10.1002/ctm2.70380

**Published:** 2025-06-27

**Authors:** Jiali Wang, Bo Qian, Xiaowen Yu, Yidan Zhang, Chunlei Zhou, Tingting Yang, Le Xia, Gang Zhang, Yi‐Xuan Zhang, Yaping Wang, Yongjun Fang

**Affiliations:** ^1^ Department of Hematology and Oncology Children's Hospital of Nanjing Medical University Nanjing China; ^2^ Department of Cardiothoracic Surgery Children's Hospital of Nanjing Medical University Nanjing China; ^3^ Inflammation and Immune Mediated Diseases Laboratory of Anhui Province, School of Pharmacy Anhui Medical University Hefei China; ^4^ Department of Pathology Children's Hospital of Nanjing Medical University Nanjing China; ^5^ School of Tianyuan Honors Nanjing Medical University Nanjing China; ^6^ Medical Basic Research Innovation Center for Cardiovascular and Cerebrovascular Diseases, Ministry of Education, China School of Pharmacy, Nanjing Medical University Nanjing China; ^7^ Department of Neurology Children's Hospital of Nanjing Medical University Nanjing China; ^8^ International Joint Laboratory for Drug Target of Critical Illnesses, School of Pharmacy Nanjing Medical University Nanjing China; ^9^ Northern Jiangsu Institute of Clinical Medicine The Affiliated Huaian No. 1 People's Hospital of Nanjing Medical University Huaian China

**Keywords:** T‐cell lymphoblastic lymphoma, hypermethylation, mediastinum preference, T‐cell differentiation stage

## Abstract

**Background:**

The clinical guideline classifies T‐LBL and T‐ALL jointly, differentiating them merely by the bone marrow blast cell proportion. However, their distinct clinical manifestations, genetic profiles, and specific pathogenic requirements have prompted us to reevaluate the differences between them.

**Methods and Results:**

We established the NCH‐TALL‐LBL cohort, which includes flow cytometry data and somatic mutation data from our center. Additionally, we collected T‐LBL samples and implemented single‐cell RNA sequencing and single‐cell T‐cell receptor sequencing. Combining the single‐cell RNA sequencing data of T‐ALL, expression array data, flow cytometry data, we discovered that malignant T cells in T‐LBL are predominantly in the DN‐ and DP‐stage blocking modes (DP cells dominate). This block mode in T‐LBL generates signals that drive the development of an immunosuppressive microenvironment and the mediastinum preference. Additionally, E2F2, an active transcription factor in the DP and DN stages, upregulates the expression of UHRF1, resulting in hypermethylation of tumor suppressor genes. Findings from in vivo and in vitro research clearly show that demethylation therapy targeting this mechanism effectively inhibits tumor proliferation in T‐LBL.

**Conclusion:**

From the perspective of differentiation blockage, T‐LBL and T‐ALL represent different stages of the same disease, and the stage block bias of T‐cell contributes to their heterogeneity.

**Key points:**

- Malignant T cells in T‐LBL are primarily blocked in the DN and DP stages, which contributes to the immunosuppressive TME and mediastinum preference of T‐LBL.
- The active transcription factor E2F2 in the DP and DN stages upregulates UHRF1 expression, leading to the hypermethylation of tumor suppressor genes in T‐LBL.
- Demethylation therapy targeting the hypermethylation of tumor suppressor genes mediated by UHRF1 effectively inhibits tumor proliferation in T‐LBL.

## Graphical abstract

- Malignant T cells in T‐cell lymphoblastic lymphoma (T‐LBL) are predominantly blocked in double negative (DN) and double positive (DP) stages.
- Block bias induces an immunosuppressive tumour microenvironment (TME) and mediastinal preference.
- Block bias upregulates UHRF1 leading to hypermethylation of tumour suppressor genes.
- Demethylation therapy exhibits potential in the treatment of T‐LBL.

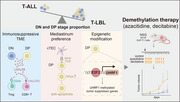

## 1 INTRODUCTION

T‐cell lymphoblastic lymphoma (T‐LBL), which emanates from precursor lymphoblastic cells, is a rare but aggressive malignancy. In terms of morphology and cellular origin, it exhibits overlapping biological features with T‐cell acute lymphoblastic leukaemia (T‐ALL). According to the clinical guideline,^1^ T‐LBL and T‐ALL are grouped under the same classification, T‐LBL is typically differentiated from T‐ALL by the bone marrow (BM) infiltration by blast cells that does not exceed 25%. However, the long‐term outcome for patients with relapsed/refractory disease remains bleak (<10%–30%).^2^ Owing to insufficient comprehension of the distinctive biological processes specific to T‐LBL has impeded the development of strategies aimed at preventing relapse, mitigating treatment toxicity, refinement of risk‐based patient stratification and devising novel therapeutic approaches.^3^

Genetic driver mutations and differentially expressed genes (DEGs) indicate that T‐LBL and T‐ALL may possess distinct pathogenic mechanisms and rely on unique molecular determinants.^4^ *NOTCH1* and/or *FBXW7* mutations suggest a positive outcome in paediatric patients with T‐LBL^5^; conversely, heterozygosity loss in the chromosomal region 6q14‐24^6^ and *PTEN* mutations^7^ are correlated with a greater risk of relapse. DEGs associated with two disease entities participate in processes such as homotypic cell–cell adhesion and chemotactic response, which may explain why T‐LBL cells tend to localise within lymphoid tissues, closely associated with stromal cells.^8^, ^9^ Although we acknowledge the differences between these two disease entities, the fragmented understanding of these differences has not yet enabled a systematic interpretation.

Paediatric cancers related to cell differentiation and tissue development,^10^, ^11^ epigenetic modifications are crucial for T‐cell differentiation, lineage commitment and maintenance of functional cells.^12^ T‐LBL and T‐ALL both arise from T‐lineage cells, their blocked differentiation stage defines the immunophenotype.^13^ Specifically, multipotent blood progenitors (MPP) and common lymphoid precursor (CLP) originate in the BM. These cells subsequently migrate to thymus, then undergo a stepwise differentiation process. The differentiation trajectory encompasses the formation of T‐cell precursor (DN (early)), double negative (DN), double positive (DP) and CD4/CD8^+^ single positive (SP) T cell. Despite research efforts that have identified disparities in immunophenotypes between T‐LBL and T‐ALL, their impact on clinical phenotypes remains unclear.^4^, ^14^, ^15^ During T‐cell development, DNA methylation is tightly regulated, but some T‐ALL patients exhibit abnormal hypermethylation of CpG islands.^16^, ^17^ Given the link between epigenetic modifications and T‐cell differentiation, we focused on epigenetic modification‐related genes in T‐LBL.

In this work, to enhance our understanding of T‐LBL, we procured T‐LBL specimens and conducted single‐cell RNA sequencing (scRNA‐seq) and single‐cell T‐cell receptor sequencing (scTCR‐seq) and compared them with T‐ALL samples. Our flow cytometry data from the NCH‐TALL‐LBL cohort revealed that T‐LBL has a bias towards blocking at the DP and DN stages, leading to a propensity to express LGALS9 and a reliance on major histocompatibility complex (MHC) signalling from thymic epithelial cells (TECs), facilitating the formation of an immunosuppressive microenvironment with a mediastinal preference. Notably, the active E2F2 transcription factor in these stages upregulated UHRF1, enhancing the methylation of tumour suppressor genes. Furthermore, in vivo and in vitro experiments experimental findings validated the responsiveness of T‐LBL to demethylation therapy.

## 2 RESULTS

### 2.1 Single‐cell transcriptomic and TCR profiling of T‐LBLs

**FIGURE 1:**
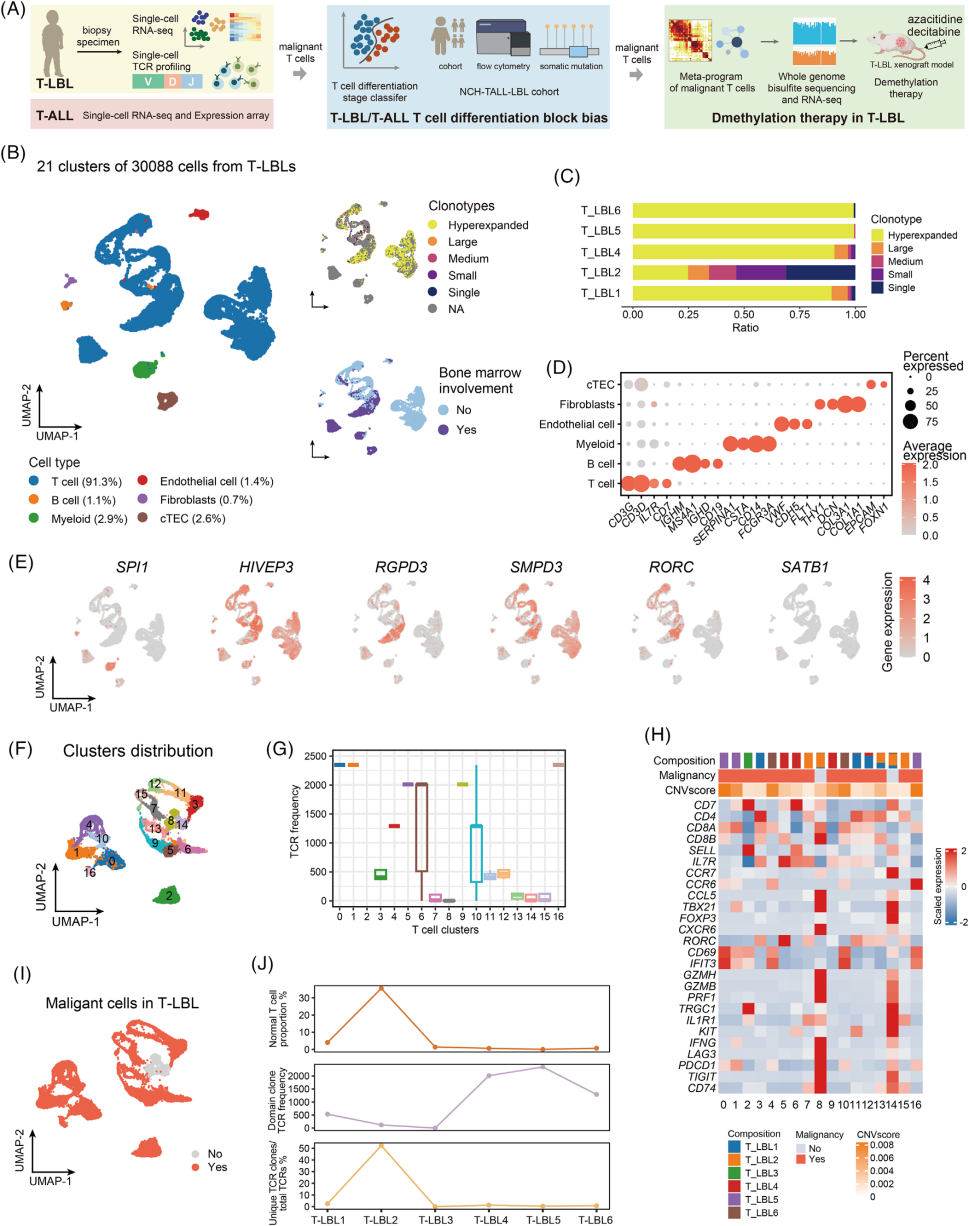
Single‐cell profiling of T‐cell lymphoblastic lymphoma (T‐LBL). (A) Workflow outlining the processes of sample collection, data analysis and validation. (B) Two‐dimensional visualisation of the annotated major cell types from T‐LBL scRNA‐seq data via uniform manifold approximation and projection (UMAP). Additional information is provided in the two subplots on the right: T‐cell receptor (TCR) clone frequency (top) and bone marrow (BM) involvement (bottom). (C) Bar plot depicting the TCR clonotype composition of each T‐LBL sample represented in B. (D) Dot plot illustrating the average expression of canonical markers in each cell type, as shown in B. (E) Expression levels of T‐cell differentiation stage‐specific genes in scT‐LBLs. (F) Unsupervised clustering of T cells derived from scT‐LBLs. (G) Boxplot representing the distribution of the TCR frequency in each T‐cell cluster of scT‐LBLs. (H) Heatmap indicating the expression levels of T‐cell function genes in each T‐cell cluster of scT‐LBLs, with patient composition, malignant status and copy number variation (CNV) scores displayed in the annotated columns. (I) Distribution of malignant and normal T‐cell clusters from scT‐LBLs in UMAP. (J) Relationships among the normal T‐cell proportion, domain clone TCR frequency and scaled unique TCR clones in T‐LBLs.

We performed scRNA‐seq and scTCR‐seq on T‐LBL samples obtained from patients diagnosed with T‐LBL via pathology and flow cytometry (Figure 1A). The comprehensive demographic information is presented in Table S1. As shown in Figure 1A, to compare T‐LBL and T‐ALL, we initiated the NCH‐TALL‐LBL cohort, which contains flow cytometry data and somatic mutation data from our centre. Additionally, we incorporated two scRNA‐seq datasets of T‐ALL, named scT‐ALLs, which included TALL1‐TALL10 from GSE227122^18^ and Pre‐TALL1 and Pre‐TALL2 from GSE132509^19^; the details of the datasets used are shown in Table S2.

After quality control of the scT‐LBLs (Figure S1A), 30 088 cells with 28 226 features were retained. Subsequently, 21 cell clusters were obtained through dimensionality reduction, with T‐cell receptor (TCR) clone frequencies ranging from 1 to 2346 (Figures 1B and S1B). Except that T‐LBL3 did not detect TCRαβ chains, there was a highly expanded TCR clone in all the other samples, which should correspond to the dominant clone in each sample (Figure 1C). Cell types were identified using canonical markers, encompassing T cells, B cells, myeloid cells, fibroblasts, endothelial cells and cortical thymic epithelial cells (cTECs) (Figure 1D).

To determine the blocked features of malignant cells, we investigated the expression levels of genes specific to different differentiation stages in scT‐LBLs (Figure 1E). The marker genes associated with the DN stage (*HIVEP3*, *RGPD3*) and DP stage (*SMPD3*, *RORC*) were highly expressed across all the samples, whereas the DN (early) marker (*SPI1*) and mature T‐cell marker (*SATB1*) presented lower activity, indicating that both the DN‐ and DP‐stage blocks were evident in T‐LBL. To define the characteristics of normal and malignant T cells, we distinguished the 17 subclusters of T cells (Figure 1F). Despite considerable heterogeneity among the T‐cell subclusters across samples, clusters 8 and 14 were consistently present in all samples (Figure S1C), the TCR frequency distribution suggested that clusters 8 and 14 did not contain hyperextended clonotypes (Figure 1G). We subsequently conducted copy number variation (CNV) analysis and evaluated the expression of functional genes, and the results also indicated that clusters 8 and 14 were normal T cells (Figures 1H and S1D). Therefore, normal and malignant T cells in scT‐LBLs were identified (Figure 1I). The proportion of normal T cells is inversely related to the dominant clone's TCR frequency and is directly related to TCR abundance (Figure 1J).

### 2.2 Differentiation and functional differences of malignant T cells in T‐LBL and T‐ALL

**FIGURE 2:**
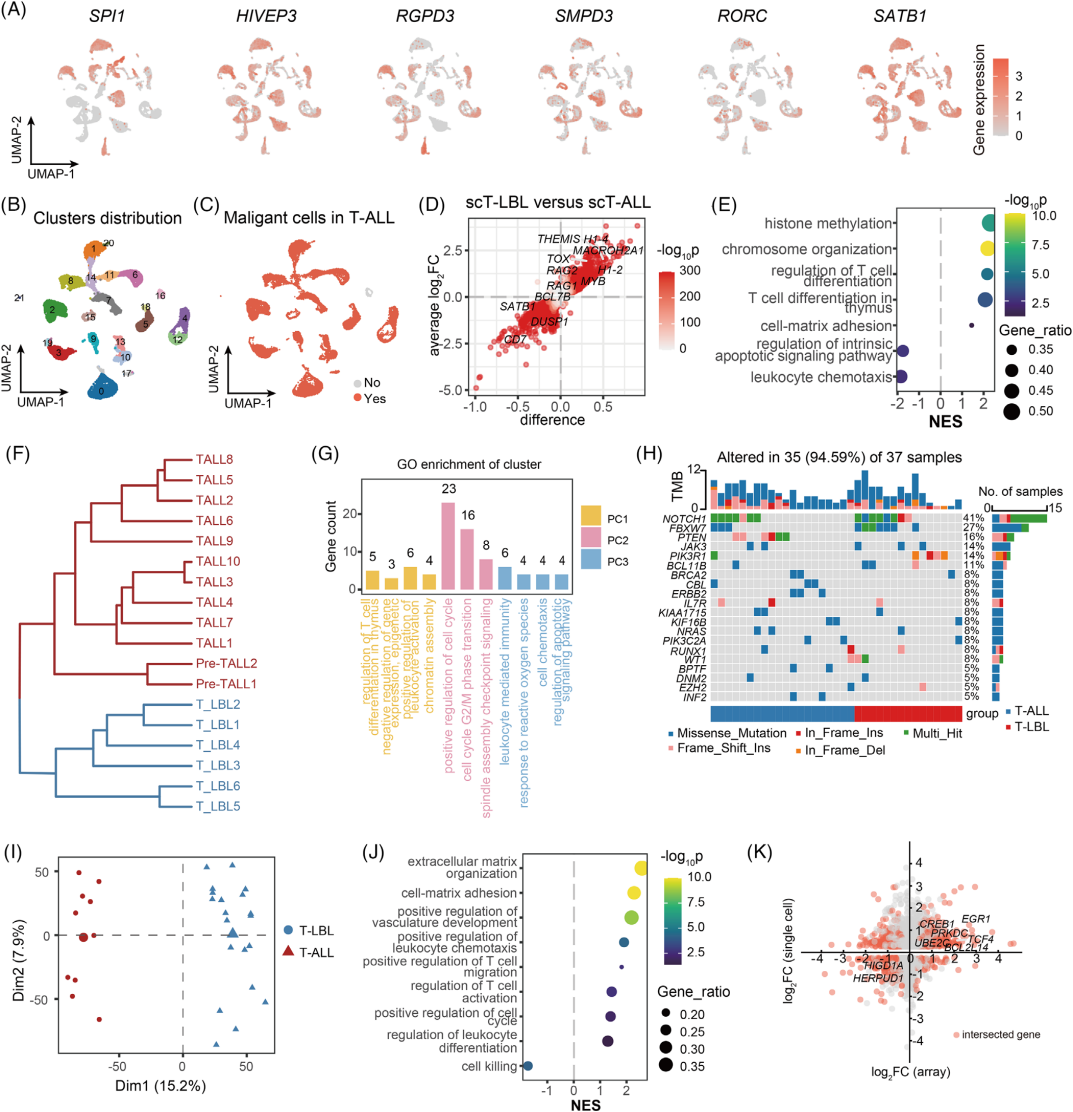
Preliminary comparison between T‐cell lymphoblastic lymphoma (T‐LBL) and T‐cell acute lymphoblastic leukaemia (T‐ALL). (A) Expression levels of T‐cell differentiation stage‐specific genes in scT‐ALLs. (B) Unsupervised clustering of T cells from scT‐ALLs. (C) Distribution of malignant and normal T‐cell clusters from scT‐ALLs in UMAP. (D) Differentially expressed genes (DEGs) of malignant T cells from scT‐LBLs and scT‐ALLs, with red dots indicating DEGs. (E) The different pathways enriched by gene set enrichment analysis (GSEA) between scT‐LBLs and scT‐ALLs. (F) Unsupervised hierarchical clustering of malignant T cells from scT‐LBLs and scT‐ALLs based on transcriptome similarity. (G) Biological functions enriched by gene ontology (GO) analysis of the first three principal components (PCs), which were used to evaluate the transcriptome similarity presented in F. (H) Comparison of somatic mutation frequencies between T‐ALL and T‐LBL based on data from the NCH‐TALL‐LBL cohort. (I) Two‐dimensional visualisation and clustering of expression array data from 20 T‐LBLs and 10 T‐ALLs in GSE29986 using principal component analysis (PCA) reduction. (J) The different pathways enriched by GSEA between T‐LBL and T‐ALL from GSE29986. (K) Intersection of DEGs from GSE29986 and DEGs from scRNA‐seq.

To investigate differences between T‐ALL and T‐LBL patients, we included the scT‐ALL dataset (12 T‐ALL BM samples). Here, 33 clusters from a total of 29 769 cells with 18 883 features were identified and annotated with markers (Figure S2A–C). T‐cell differentiation stage‐specific genes were examined in scT‐ALLs (Figure 2A). Compared to T‐LBL, the expression of DN (early) stage genes and mature stage genes were higher in T‐ALL. Thus, blocked differentiation stage of T cells may vary in these two entities. Malignant T cells in scT‐ALLs were identified, among all 22 T‐ALL cell subclusters (Figure 2B), clusters 14 and 16 were consistently observed across patients (Figure S2D), the CNV scores and T‐cell function gene expression data indicated that cluster 16 was normal T cells (Figure S2E,F), whereas the others were malignant T cells (Figure 2C). In both scT‐LBLs and scT‐ALLs, malignant T cells predominated (Figure S2G).

We carried out DEG analysis on malignant T cells from scT‐LBLs and scT‐ALLs and identified 3096 upregulated and 1100 downregulated genes in scT‐LBLs (Figure 2D and Table S3). The gene set enrichment analysis (GSEA) was employed to detect significantly altered biological processes in T‐LBL, and the normalised enrichment score (NES) was utilised to evaluate changes (Figure 2E). Our analysis revealed that T‐cell differentiation was enhanced and that the regulatory pathways for apoptosis were weakened, indicating that the cell differentiation stages of malignant T cells are different. Transcriptome similarity analysis revealed stable differences between scT‐LBL and scT‐ALL via hierarchical clustering (Figure 2F). Gene ontology (GO) analysis of the first three principal components (PCs) of the transcriptome similarity assessment revealed that the main differences in chromatin assembly and epigenetic modification, cell cycle regulation and apoptosis (Figure 2G and Table S4), elucidates the unique biological processes of malignant T cells in T‐LBL and suggests that differences in T‐cell differentiation may be related to these processes.

To deepen understanding in a larger cohort, we analysed the somatic mutation data from the NCH‐TALL‐LBL cohort. However, we did not observe any significant differential mutations between these two disease entities (Figure 2H and Table S5). We then conducted a principal component analysis (PCA) on the expression array dataset GSE299868,^8^ containing 20 T‐LBL samples and 10 T‐ALL samples. The results revealed that T‐LBL and T‐ALL samples were clearly segregated along PC1 axis (Figure 2I). In T‐LBL, we identified 1799 upregulated and 1181 downregulated DEGs compared with those in T‐ALL (Figure S2H and Table S6). Moreover, the results of GSEA were related to the extracellular matrix organisation, leukocyte differentiation and cell killing (Figure 2J). Genes linked to T‐cell differentiation, cell cycle transition and apoptosis regulation pathways, such as *EGR1, UBE2C* and *BCL11B*, presented consistent expression patterns between the scRNA‐seq and array datasets (Figure 2K).

### 2.3 More malignant T cells are blocked at the DP and DN stages in T‐LBL compared to T‐ALL

**FIGURE 3:**
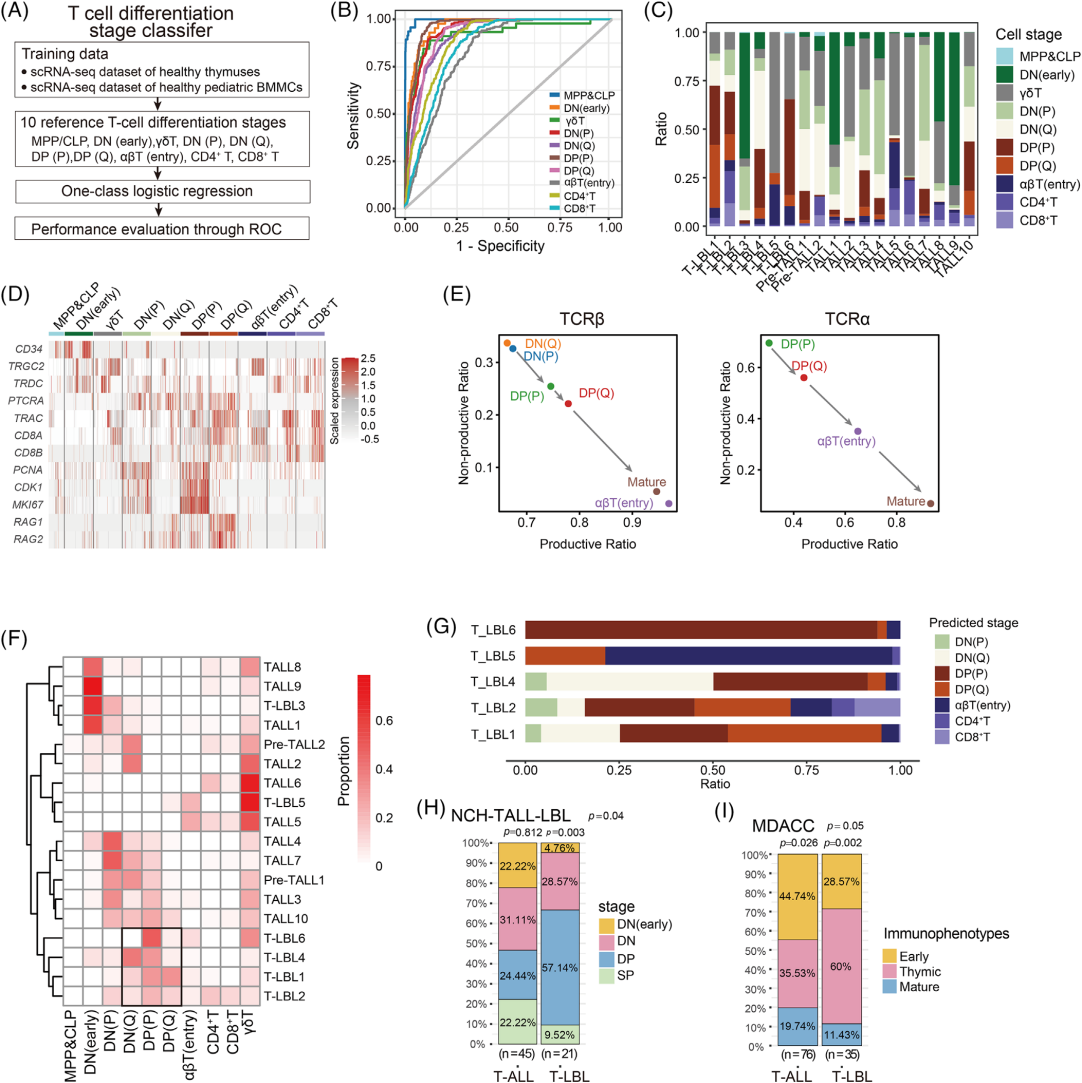
T‐cell differentiation stage block bias in T‐cell lymphoblastic lymphoma (T‐LBL). (A) Workflow for the construction of the T‐cell differentiation stage classifier. (B) Receiver operating characteristic (ROC) curves illustrating the performance of T‐cell differentiation stage classifier. (C) Composition of 10 differentiation stages in each sample from scT‐LBLs and scT‐ALLs. (D) Heatmap indicating the expression levels of hallmark genes at each predicted differentiation stage by down‐sampling to 200 cells per stage of T cells from scT‐LBLs and scT‐ALLs. (E) Proportion of productive and non‐productive T‐cell receptor (TCR)β and TCRα chains at each differentiation stage of scT‐LBLs. (F) Heatmap showing the proportion of each T‐cell differentiation stage in scT‐LBLs and scT‐ALLs, with clustering results indicating T‐LBL block bias. (G) Composition of T‐cell differentiation stages within the dominant TCR clone of each T‐LBL sample. (H) Flow cytometry data of T‐LBL and T‐ALL patients from our centre. *p* values were calculated via Fisher's exact tests. (I) Immunophenotype distribution of T‐LBL and T‐ALL data from the University of Texas MD Anderson Cancer Center (MDACC). *p* values were calculated by Fisher's exact tests.

Our analysis uncovered the differentiation stage block of T cells is the main distinction between these two T‐lineage tumours, and we developed a T‐cell differentiation stage classifier to further detail the block stage composition (Figure 3A and Table S7). This classifier was trained using two additional datasets: healthy thymus samples (E‐MTAB‐8581^20^) and paediatric BM mononuclear cells (GSE132509^19^). We extracted the following 10 T‐cell differentiation stages from these datasets: CLP/MPP, DN (early), γδT, DN (P) (proliferating DN), DN (Q) (quiescent DN), DP (P) (proliferating DP), DP (Q) (quiescent DP), αβT (entry), CD4^+^T and CD8^+^T cells. These annotated stages were utilised to train the classifier, which accurately identified the T‐cell differentiation stages (Figures 3B and S3A–I).

Using this classifier, we assessed the composition of malignant T‐cell differentiation stages in scT‐LBLs and scT‐ALLs (Figure 3C) and presented the expression of hallmark genes in a heatmap (Figure 3D). The stem cell marker *CD34* was predominantly expressed at the DN (early), followed by the upregulation of genes related to the TCRγδ chains in some DN (early) and γδT cells. Furthermore, the dynamic expression trends of *PTCRA*, *TRBC* and *TRAC* aligned with T‐cell maturation, progressing from DN (P) and DN (Q) to CD4^+^ and CD8^+^T cells.

We conducted an analysis of the scTCR‐seq to monitor the modifications in TCRαβ chains across various developmental stages in T‐LBL (Figure 3E). TCRα chains were first detected at the DP stage, with a rising productive ratio from the DP (P) stage to the SP stage. A comparable pattern was noted for the TCRβ chains. The malignant T cells of T‐LBL3 were blocked in the DN (early) stage, both TCRαβ chains were absent, and the scTCR‐seq data were undetectable. This confirms the accuracy of the differentiation stage prediction, as the TCRαβ chain features match the predicted stages.

Next, we clustered samples by the predicted differentiation stage proportions to identify block features of T‐LBL and T‐ALL (Figure 3F). The results revealed that malignant T cells exhibited several developmental block modes, spanning from the DN (early) stage through the DN stage, reaching the DP stage and potentially extending to the more mature SP or γδT stage. Notably, the T‐LBL samples exhibit as DN‐ and DP‐stage blocking mode (DP cells dominated), as indicated in the highlighted area by the black frame. We also calculated the fraction of differentiation stages for the dominant clone in each T‐LBL sample on the basis of the TCR frequency (Figure 3G), identifying DN (Q), DP (P) and DP (Q) as the main differentiation stages.

To further validate the distributions of T‐cell differentiation stage block, we analysed flow cytometry data from the NCH‐TALL‐LBL cohort (Tables S2 and S8), and a significantly higher proportion of T‐LBL samples were blocked at the DP stage than were T‐ALL samples (Figure 3H). Furthermore, we analysed the data from the University of Texas MD Anderson Cancer Center (MDACC) based on the work of Jain et al.^21^ These data adhere to the 2008 version of the WHO Classification of Tumours of Haematopoietic and Lymphoid Tissues; according to this classification standard, most thymic immunophenotype samples correspond to T cells blocked in the DP stage. Figure 3I shows that a greater proportion of T‐LBL samples displayed a thymic immunophenotype, further supporting the view that T cells in T‐LBL tend to be blocked at the DP differentiation stage.

### 2.4 Mediastinum preference of T‐LBL resulting from the malignant T‐cell differentiation stage block bias

T‐cell differentiation is rigorous, during DP cell development in the thymus, these cells undergo positive selection, a mechanism guarantees the survival of DP cells that can effectively recognise self‐MHC molecules presented by TECs.^22^ This suggests that the high proportion of DP cells in T‐LBL may indicate an essential role for MHC signals from TECs in mitigating apoptosis during T‐LBL development.

**FIGURE 4:**
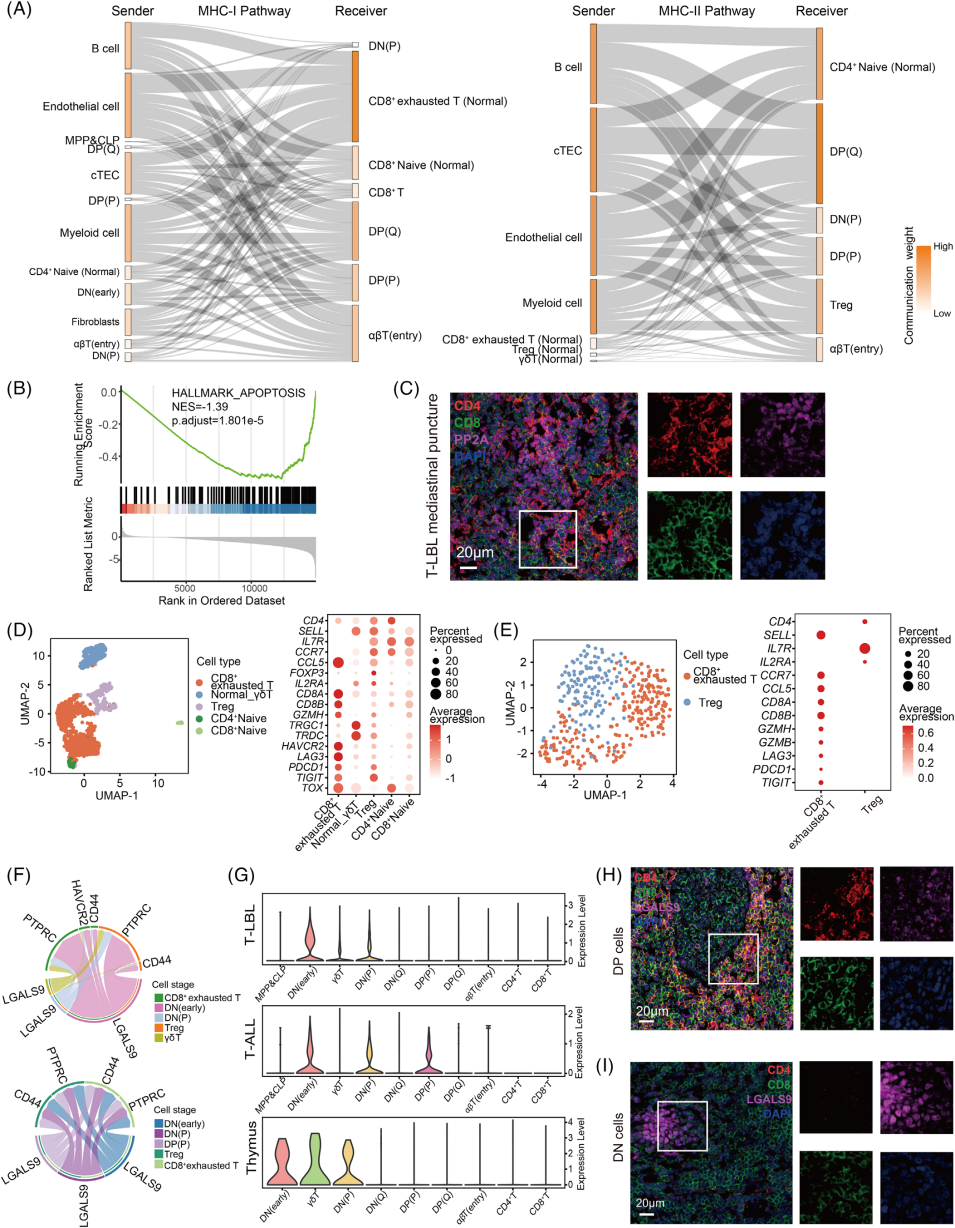
Mediastinum preference of T‐cell lymphoblastic lymphoma (T‐LBL) involving major histocompatibility complex (MHC) signals and LGALS9 signals. (A) Sankey diagram illustrating the communication weights of the MHC‐I (left) and MHC‐II (right) pathways between source and target cells in T‐LBL. (B) Comparison of apoptotic pathway enrichment in DP cells from T‐LBL and healthy thymus samples via GSEA. (C) Representative images of multicolour immunofluorescence staining of CD4, CD8 and PP2A in mediastinal puncture of T‐LBL. (D) Normal T cells subclustered and annotated in scT‐LBLs (left), with a dot plot representing the average expression of canonical markers in each cell type (right). (E) Normal T cells subclustered and annotated in scT‐ALLs (left), with a dot plot representing the average expression of canonical markers in each cell type (right). (F) Source and target cells of the GALECTIN pathway in scT‐LBLs (top) and scT‐ALLs (bottom). (G) Expression specificity of LGALS9 in T cells at each differentiation stage from scT‐LBLs, scT‐ALLs and thymus cells from healthy thymus samples. (H) Representative images of CD4, CD8 and LGALS9 multicolour immunofluorescence staining of DP cells from mediastinal puncture of T‐LBL. (I) Representative images of CD4, CD8 and LGALS9 multicolour immunofluorescence staining of double negative (DN) cells from mediastinal puncture of T‐LBL.

To verify this hypothesis, we analysed cell communication in the scT‐LBLs and thymus dataset (including thymus cells and TECs from sample T07). The communication weights are illustrated in Figure S4A,B. We concentrated on the interaction intensity within MHC pathways between T cells and TECs and compared these communication relationships in Figures 4A and S4C; robust communication signalling between DP cells and cTECs is shown. The presence of survival signals contributed to the attenuation of the cell apoptosis process in the DP cells from the T‐LBL group compared with those from the healthy thymus samples according to GSEA (Figure 4B). Given that PP2A serves as a downstream antiapoptotic signal activated by the MHC pathway in DP cells,^23^ we assessed PP2A expression in mediastinal punctures from T‐LBL patients (Figure 4C). As shown in the magnified immunofluorescence image, the intracellular intensity of PP2A in CD8^+^T cells located at the lower left corner was markedly less than that in DP cells at the upper right corner, indicating the need for antiapoptotic signals in DP cells.

The tumour microenvironment (TME) harbours normal T cells, particularly Tregs and CD8^+^ exhausted T cells (Figure 4D,E). According to the scTCR‐seq data, single and small clones were the main clonotypes (Figure S4D), along with the expression of *LAG3*, *PDCD1* and *TIGIT*, indicating an immunosuppressive TME. Our analysis identified a GALECTIN pathway present in these two T‐lineage tumours (Figure 4F), LGALS9 signals emitted from DN and DP cells interact with the HAVCR2 receptor on CD8^+^T cells and the CD44 receptor on Tregs. LGALS9‐HAVCR2 induces apoptosis and proliferation arrest in CD8^+^T cells,^24^ and LGALS9‐CD44 promotes the maintenance and functionality of Tregs.^25^ Moreover, scRNA‐seq data revealed that *LGALS9* was expressed in DN, DP and γδT cells (Figure 4G).

We subsequently analysed LGALS9 expression in the mediastinal punctures of T‐LBL patients. As shown in the magnified immunofluorescence image in Figure 4H, the intensity of LGALS9 in CD8^+^T cells located at the upper left corner was lower than that in DP cells at the lower right corner. Furthermore, as shown in the magnified immunofluorescence image in Figure 4I, the intensity of LGALS9 in DN cells on the left side was notably greater than that in CD8^+^T cells on the right side. Consequently, elevated LGALS9 signals from DN and DP cells contribute to the immunosuppressive microenvironment. Collectively, these findings indicate that the MHC and LGALS9 signals induced by DP and DN cells render the mediastinum a favourable niche for T‐LBL development.

### 2.5 Malignant T cells blocked in the DP and DN stages exhibit activation of DNA replication and epigenetic regulation

**FIGURE 5:**
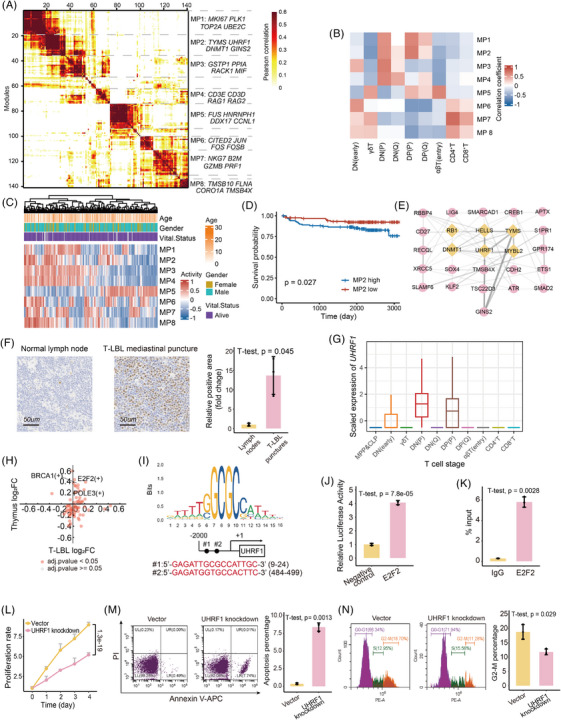
Active meta‐programs in the double negative (DN) and double positive (DP) stages. (A) Heatmap showing the clustering results of 141 gene expression programs derived from non‐negative matrix factorisation (NMF) analysis of T‐cell lymphoblastic lymphoma (T‐LBL) and T‐cell acute lymphoblastic leukaemia (T‐ALL); eight active meta‐programs (MPs) are presented, along with top‐scoring gene annotations. (B) Heatmap illustrating the Pearson correlation coefficients between MP activity and the proportions of 10 T‐cell differentiation stages in scT‐LBLs and scT‐ALLs. (C) Heatmap of MP activity in each sample of the TARGET‐ALL‐P2 cohort. Patient age, gender and vital status are indicated in the annotation rows. (D) Kaplan–Meier curves representing overall survival for the high‐ and low‐MP2 groups, divided by the median value. *p* values were calculated using the log‐rank test. (E) Protein–protein interaction (PPI) network of the top‐scoring genes in MP2, with yellow nodes representing hub genes. (F) Representative immunohistochemistry images of UHRF1. Bar plot indicating the relative positive area of UHRF1 in mediastinal punctures of T‐LBLs and normal lymph nodes. The data are presented as the means ± SDs, and *p* values were calculated by two‐tailed *t*‐test. (G) Boxplot indicating that UHRF1 is highly expressed in the DN and DP stages of T cells from scT‐LBLs. (H) Scatter plot indicating the intersection of differentially active transcription factors in thymus cells from healthy thymus samples and scT‐LBLs; differential fold changes were calculated as the activity of the DN and DP stages compared with that of other stages. (I) Potential binding sites of E2F2 in the promoter region of UHRF1 predicted by JASPAR database. (J) Bar plot indicating the dual‐luciferase activity of the negative control and E2F2 overexpression groups, demonstrating that E2F2 can regulate UHRF1. The data are presented as the means ± SDs, and *p* values were calculated by two‐tailed *t*‐test. (K) Bar plot indicating the fold difference in the UHRF1 promoter region enriched by the anti‐E2F2 antibody than the control IgG antibody. The result of ChIP‐qPCR indicating the E2F2 binding to promoter region of UHRF1. The data are presented as the means ± SDs, and *p* values were calculated by two‐tailed *t*‐test. (L) Proliferation curves of vector control and UHRF1‐knockdown cells, demonstrating that the proliferation rate of UHRF1‐knockdown cells is significantly slower than that of vector‐treated cells. The data are presented as the means ± SDs, and *p* values were calculated by one‐way ANOVA. (M) Flow cytometry analysis apoptosis of vector‐ and UHRF1‐knockdown cells, along with barplot of apoptosis percentage of vector‐ and UHRF1‐knockdown cells. The data are presented as the means ± SDs, and *p* values were calculated by two‐tailed *t*‐test. N. Flow cytometry analysis of cell distribution across different cell cycle phases, along with bar plot of the proportion of cells in the G2–M phase in vector‐ and UHRF1‐knockdown cells. The data are presented as the means ± SDs, and *p* values were calculated by two‐tailed *t*‐test.

To explore the biological effects associated with T‐cell differentiation stage block bias, 141 distinct gene expression programs were dissected from the malignant T cells of scT‐LBLs and scT‐ALLs, and subsequently clustered into eight meta‐programs (MPs; Figure 5A). The functions of each MP are reflected by the top‐scoring genes (Table S9). For example, MP1 is related to the cell cycle and proliferation, and MP2 is linked to DNA replication and epigenetic regulation.

We subsequently calculated Pearson correlations between the MPs and T‐cell differentiation stages to pinpoint the active stages of each MP. The correlation heatmap of Figure 5B shows that MP1, MP2 and MP4 are predominantly active at the DP and DN stages. Owing to the absence of a T‐LBL cohort that includes both RNA‐seq and survival data, we employed the TARGET‐ALL‐P2 cohort to elucidate the effects of MPs on survival. Gene set variation analysis (GSVA) indicates MPs’ activity levels (Figure 5C), and patients exhibiting higher MP2 activity generally experienced poorer overall survival outcomes (Figures 5D and S5A).

### 2.6 UHRF1 is upregulated and mediates the proliferation of malignant T cells in T‐LBL

To further investigate specific mechanisms by which MP2 is associated with DP/DN stages and poorer outcomes, the protein–protein interaction (PPI) network within MP2 was constructed to identify hub proteins (Figure 5E and Table S10). Among the six hub proteins, UHRF1 has emerged as an epigenetic modulator that is highly expressed, specifically in the thymus (Figure S5B,C). This distinctive expression pattern indicates that UHRF1 could have a pivotal function in T‐cell differentiation. Immunohistochemical analyses revealed that UHRF1 expression levels in mediastinal punctures samples from patients with T‐LBL were markedly higher than those in lymph nodes of healthy individuals (Figures 5F and S5D). Additionally, both malignant T cells and normal thymus cells at the DN (P) and DP (P) stages presented elevated *UHRF1* expression (Figures 5G and S5E).

**FIGURE 6:**
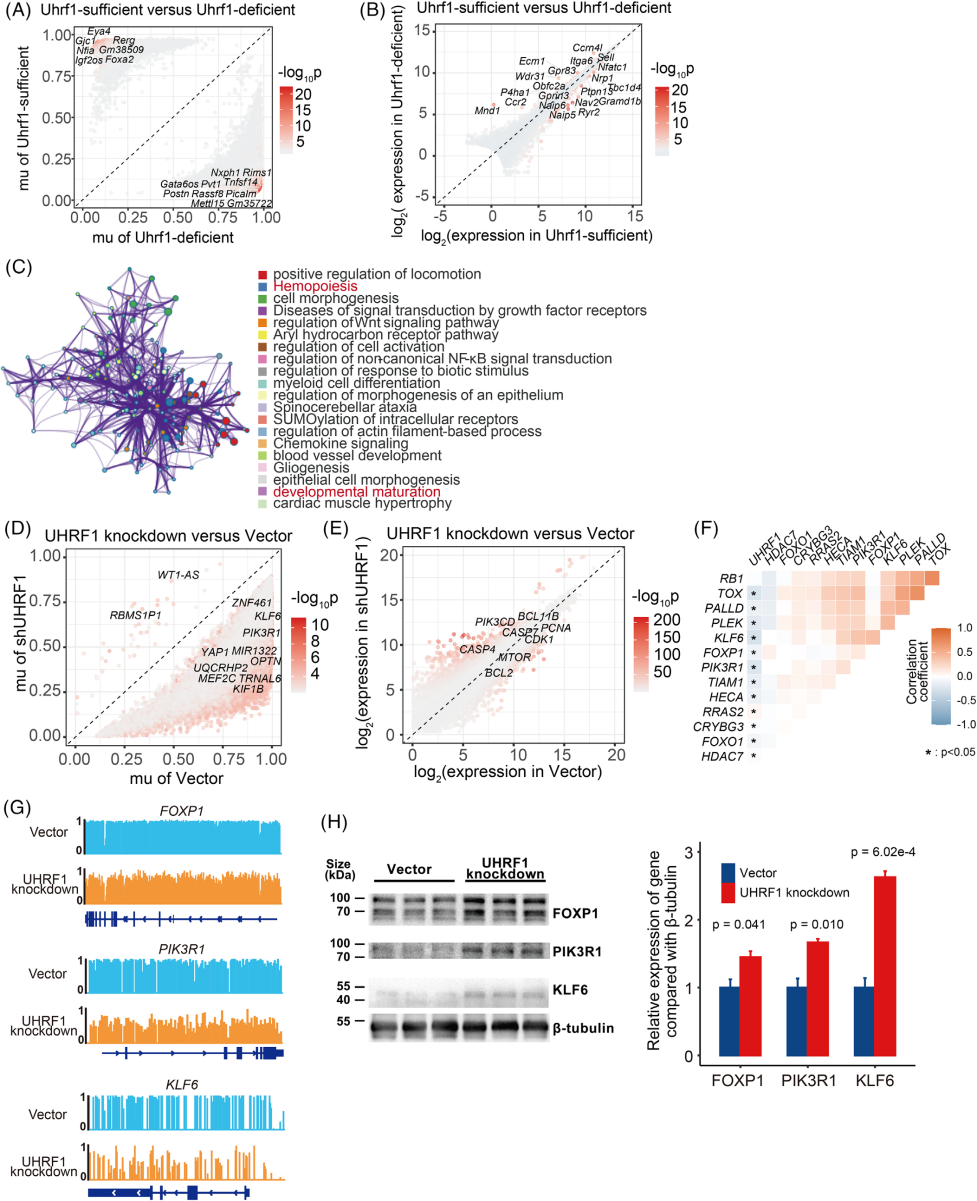
UHRF1 affects the malignancy of T‐cell lymphoblastic lymphoma (T‐LBL) through DNA methylation. (A) Scatter plot indicating differentially methylated loci (DMLs) in Uhrf1‐sufficient and Uhrf1‐deficient T cells, with red dots representing the DMLs. (B) Scatter plot displaying DEGs in Uhrf1‐sufficient and Uhrf1‐deficient T cells, with red dots representing the DEGs. (C) Metascape network enrichment analysis of intersected DMLs and DEGs, displaying the top 20 enriched terms; dot colours indicate different enriched terms. (D) Scatter plot indicating differentially methylated regions (DMRs) in vector‐ and UHRF1‐knockdown cells, with red dots representing the DMRs. (E) Scatter plot indicating DEGs in vector‐ and UHRF1‐knockdown cells, with red dots representing the DEGs. (F) Heatmap illustrating the Pearson correlation between target downstream genes and UHRF1 in the scT‐LBLs. (G) Methylation profile of the target genes of UHRF1 in vector control and UHRF1‐knockdown cells, namely, *FOXP1*, *PIK3R1* and *KLF6*. H. Verification of target proteins of UHRF1 by WB analysis in vector‐ and UHRF1‐knockdown cells. FOXP1, PIK3R1 and KLF6 were significantly upregulated following UHRF1 knockdown. The data are presented as the means ± SDs, and *p* values were calculated by two‐tailed *t*‐test.

To verify whether the high expression of UHRF1 in the DN and DP stages was regulated by T‐cell differentiation‐related transcription factors, we analysed the changes in transcription factor activity between the DN and DP stages compared with other differentiation stages via scRNA‐seq data derived from T‐LBL samples and healthy thymus samples. Notably, *E2F2* exhibited the most significant increase in activity at the DN and DP stages in both samples (Figure 5H and Table S11), and there are two potential E2F2 binding sites in the *UHRF1* promoter (Figure 5I). Through dual‐luciferase reporter assays and Chip‐qPCR experiments, we confirmed that E2F2 can upregulate the expression of UHRF1 (Figure 5J,K).

To explore the effect of UHRF1 on T‐LBL, we utilised SUP‐T1, a human T‐LBL cell line blocked at the DP stage.^26^ After *UHRF1* was knocked down in SUP‐T1 cells (Figure S6A–C), the cells exhibited a marked decrease in spheroid formation capacity (Figure S6D), and the proliferation rate also decreased significantly (Figure 5L). In contrast to those in the control, the rates of apoptosis and the extent of cell cycle arrest increased in UHRF1‐knockdown cells (Figure 5M,N). In summary, UHRF1 significantly affects the malignancy of T‐LBL.

### 2.7 Hypermethylation of tumour suppressor genes by UHRF1 enhances the malignancy of T‐LBL

Recent studies have identified UHRF1 as a crucial epigenetic regulator.^27^ Helmin et al. conducted reduced representation bisulphite sequencing (RRBS) combined with RNA‐seq on Uhrf1‐sufficient and Uhrf1‐deficient T cells (GSE143974),^28^ suggesting that Uhrf1 is crucial for DNA methylation and T‐cell differentiation regulation. Given the conserved amino acid sequence of UHRF1 (Figure S6E), we analysed the above dataset to identify differentially methylated loci (DMLs) and DEGs between Uhrf1‐sufficient and Uhrf1‐deficient T cells (Figure 6A,B and Tables S12 and S13). A total of 81 intersecting genes were identified between DMLs and DEGs (Figure S6F and Table S16), the enriched Metascape network analysis revealed the biological processes related to hemopoiesis and developmental maturation (Figures 6C and S6G).

To comprehensively investigate how UHRF1 affects T‐LBL through DNA methylation, whole genome bisulphite sequencing (WGBS) and RNA‐seq were conducted on UHRF1 knockdown and vector control cells. After knockdown *UHRF1*, the methylation levels in SUP‐T1 significantly decreased compared with those in the control (Figure S7A). Specifically, UHRF1‐knockdown cells presented 45 hyper‐differentially methylated regions (DMRs) and 43498 hypo‐DMRs (Figure 6D and Table S14), along with 2138 upregulated and 2124 downregulated DEGs (Figure 6E and Table S15). Overall, the DMRs and DEGs intersected, revealing 3090 shared genes (Figure S7B and Table S16). Different pathways were mainly enriched in the lymphocyte differentiation and T‐cell proliferation pathways (Figure S7C). Finally, 14 target genes were identified after the data of UHRF1‐knockdown cells and Uhrf1‐deficient T cells were intersected (Figure S7D and Table S16).

An analysis of the correlation between UHRF1 expression and that of its target genes in scT‐LBLs demonstrated that the tumour suppressor genes *FOXP1*, *PIK3R1* and *KLF6* exhibited a significant negative correlation with *UHRF1* expression (Figure 6F). According to the WGBS results, tumour suppressor genes were hypermethylated in control cells compared with UHRF1‐knockdown cells (Figure 6G), and the protein levels were also increased (Figure 6H). Overall, our findings suggest that UHRF1 methylates tumour suppressor genes to sustain the malignancy of T‐LBL.

### 2.8 Demethylation therapy improved T‐LBL outcomes by reversing the hypermethylation of tumour suppressor genes mediated by UHRF1

**FIGURE 7:**
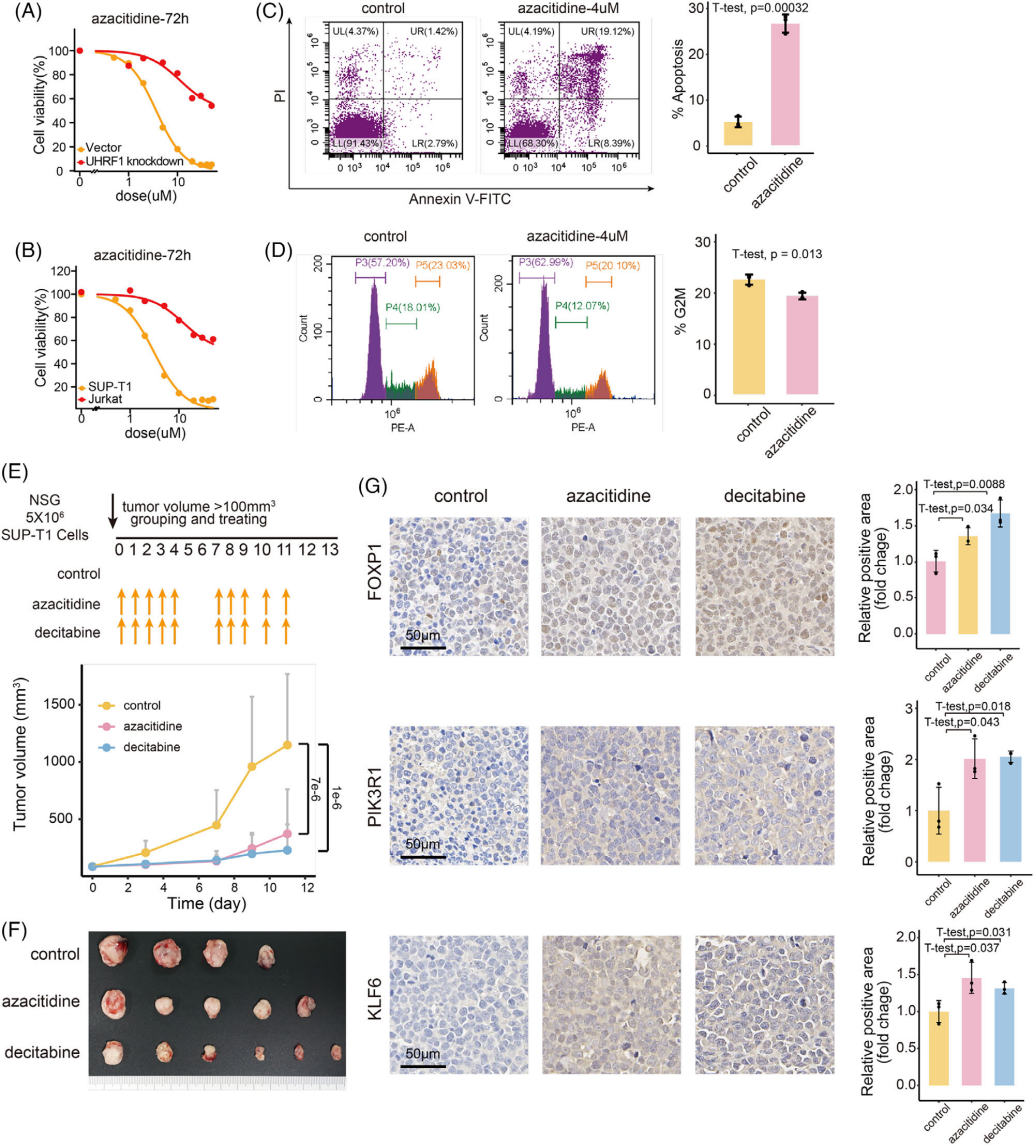
Efficacy of demethylation therapy for T‐cell lymphoblastic lymphoma (T‐LBL). (A) Comparison of azacitidine IC50 curves between UHRF1‐knockdown and vector cells. (B) Comparison of azacitidine IC50 curves between SUP‐T1 and Jurkat cells. (C) Flow cytometry analysis of the apoptosis of SUP‐T1 cells treated with 4 µM azacitidine for 72 h, along with a bar plot of the percentage of apoptotic cells in the control and azacitidine treatment groups. The data are presented as the means ± SDs, and *p* values were calculated by two‐tailed *t*‐test. (D) Flow cytometry analysis of cell distribution across different cell cycle phases, along with bar plot of the proportion of cells in the G2–M phase in the control and azacitidine treatment groups. The data are presented as the means ± SDs, and *p* values were calculated by two‐tailed *t*‐test. (E) Tumour growth curves for the control, azacitidine and decitabine treatment groups (*n* = 6) following the administration schedule shown at the top. The data are presented as the means ± SDs, and *p* values were calculated by one‐way ANOVA with Holm–Sidak's multiple‐comparison test. (F) Comparison of tumour sizes among the control, azacitidine and decitabine treatment groups. Mice whose tumours reached 1500 mm^3^ were euthanised at that time point. (G) Representative immunohistochemistry images showing changes in UHRF1 target protein expression following demethylation treatment in tumours. Bar plots indicating that the expression levels of UHRF1 target proteins in tumours were significantly upregulated following demethylation treatment. The data are presented as the means ± SDs, and *p* values were calculated by two‐tailed *t*‐test.

Given that UHRF1 decreases the expression of tumour suppressor genes in T‐LBL via DNA methylation, along with the favourable preclinical data on hypomethylating agents,^29^ we further explored the ability of demethylation therapy to restore T‐LBL tumour suppressor gene expression. In vitro, we chose azacitidine as a candidate drug for T‐LBL demethylation therapy. Dose–response curves for UHRF1 knockdown and vector control cells (Figure 7A), as well as for normal SUP‐T1 and Jurkat (single CD4^+^T cells) cells (Figure 7B), were generated after exposure to azacitidine. SUP‐T1 expressing normal levels of UHRF1 presented greater sensitivity to demethylation therapy. Subsequently, we treated SUP‐T1 cells with azacitidine. The impacts of this treatment on apoptosis and cell cycle arrest were comparable to those witnessed in UHRF1‐knockdown cells (Figure 7C,D), along with the upregulation of the UHRF1 target genes *FOXP1*, *PIK3R1* and *KLF6* (Figure S7E).

To assess the efficacy of demethylation therapy in vivo, NSG mice were inoculated subcutaneously with SUP‐T1 and divided into demethylated treatment (azacitidine and decitabine) and control groups, and the drug administration regimens are detailed at the top of Figure 7E. The tumour growth curves of the azacitidine and decitabine treatment groups were notably less steep compared to those of the control group (Figure 7E). Upon completion of the experiment, the tumours in the azacitidine and decitabine treatment groups were notably smaller than those in the control group (Figure 7F). Furthermore, immunohistochemical staining of tumour tissues was carried out to assess the expression magnitudes of proteins regulated by UHRF1 (Figures 7G and S7F). Tumour suppressor genes that were previously suppressed by UHRF1 were restored following demethylation therapy. These findings demonstrate that demethylation treatment can effectively inhibit the growth of T‐LBL.

## 3 DISCUSSION

The fifth edition of the WHO Classification of Haematolymphoid Tumours guideline categorises T‐LBL and T‐ALL together,^1^ distinguishing them exclusively according to the percentage of blasts in the BM. Currently, comparisons in these two entities have been conducted, involving mutations,^30^ methylation,^31^ CNVs^31^ and transcriptomes.^8^ We aimed to enhance the comprehension of the disparities in these two T‐lineage tumours by utilising scRNA‐seq, scTCR‐seq and WGBS, an approach that effectively addresses the heterogeneity of tumour cells and their interactions with the TME.

Our findings demonstrated significant disparities in these two entities, mainly attributable to the blocked differentiation stages of malignant T cells. In the NCH‐TALL‐LBL cohort, 12/21 of the T‐LBL patients exhibited block at the DP stage, whereas 11/45 of the T‐ALL patients did. The MDACC cohort data also revealed a greater thymic immunophenotype prevalence in patients with T‐LBL, characterised by DP‐stage block. Given the blocked differentiation stage bias, E2F2, which is recognised to be functionally active during the DN and DP stages,^20^ continues to exhibit activity in malignant T cells at these stages. E2F2 subsequently upregulates UHRF1, a protein that bridges histone modifications and DNA methylation. By investigating the role of epigenetic regulator UHRF1 and its mechanism in the methylation of tumour suppressor genes in malignant T cells, following intervention with azacitidine and decitabine, we assessed the effectiveness of demethylating therapy for T‐LBL. Therefore, during the clinical treatment, we can identify patients whose T cells are predominantly block at the DP/DN stage using flow cytometry. By integrating DNA methylation sequencing and in vitro drug sensitivity assays, we can further expand the clinical cohort to validate the differentiation stage bias of T‐LBL and its responsiveness to demethylation therapy.

Moreover, the differentiation stage block bias may shed light on the prevalence of T‐LBL in the mediastinum, as more DP cells are present in T‐LBL at the initial development stage. During the DP stage, T cells experience positive selection, where their TCRs interact with the MHC expressed by TECs.^22^ If the interaction between the TCR and MHC is either too high or too low, T cells may undergo apoptosis or be excluded from further maturation. Following TCR stimulation in DP cells, PP2A is activated and plays an antiapoptotic role; there is an abundance of PP2A in T‐LBL tissues, which supports the maintenance of this protective process.^23^ During the initiation stage of T‐LBL, this mechanism may be crucial for the survival of malignant DP cells, contributing to their mediastinum preference.

Furthermore, in both non‐malignant and malignant T cells, those in the DN, DP and γδT stages express LGALS9, an immunomodulatory ligand. By binding to HAVCR2, LGALS9 is capable of facilitating the apoptosis of CD8^+^T cells.^24^ Additionally, the immunosuppressive function of Treg is enhanced through the LGALS9‐CD44 communication pathway.^25^ Consequently, an immunosuppressive TME is established in T‐lineage tumours, which is especially prominent during the initial phases of T‐cell differentiation.

In this research, we employed multi‐omics strategies to explore the distinctions between T‐LBL and T‐ALL. From the perspective of differentiation blockage, our evidence suggests that these two entities represent different stages of the same disease. Specifically, the T cells in these two disease entities exhibit a distinct differentiation stage block distribution. This distinction shapes the biological characteristics of malignant T cells and impacts their interactions with the microenvironment. However, in our analysis, we did not focus on the unique clinical manifestations of T‐ALL and T‐LBL that arise from differential biological processes such as leukocyte chemotaxis, angiogenesis, the cell cycle and apoptosis regulation,^4^, ^8^, ^31^ which requires further study. Overall, the biological effects resulting from DNA methylation and LGALS9 secretion caused by malignant T cells are blocked at the DP and DN stages, and attention should be given to the clinical treatment for both T‐LBL and T‐ALL.

## 4 METHODS AND MATERIALS

### 4.1 Clinical data processing

The samples confirmed as T‐LBL by the pathology department were subsequently processed for scRNA‐seq and scTCR‐seq. Patients registered in our department were included, and those without complete information were excluded. In total, six T‐LBL samples, designated T‐LBL1 to T‐LBL6, were collected for scRNA‐seq and scTCR‐seq. To ascertain differentiation stages of malignant T cells in T‐LBL and T‐ALL, flow cytometry data were analysed for the following markers: CD117, CD34, CD1a, surface CD3, TCRαβ, TCRγδ, CD4 and CD8.

### 4.2 In vivo xenograft study

Prior to the experiment, 5–7‐week‐old female NSG mice were acclimatised for 7 days and then inoculated subcutaneously with SUP‐T1 cells (5 × 10^6^ cells/mouse) on the right forelimb. Upon the tumour volume attaining 100 mm^3^, the mice were randomly allocated to control group, azacitidine treatment group or decitabine treatment group. The mice in the azacitidine group were injected intraperitoneally with azacitidine (5 mg/kg) for 5 consecutive days, followed by a 2‐day drug‐free interval, constituting one cycle, which was repeated for a total of two cycles. Mice in the decitabine group were administered intraperitoneal injections of decitabine (.6 mg/kg) following the same dosing regimen as those in the azacitidine group. Mice assigned to the control group received a corresponding volume of the vehicle solution. Tumour volume was measured using callipers two to three times per week and calculated according to the formula $V=.5\times (\mathrm{lengh}\times \mathrm{widt}h^{2})$.

### 4.3 Single‐cell RNA sequencing and single‐cell T‐cell receptor sequencing

A dissociated single‐cell suspension (1 × 10^5^ cells/mL) was loaded into the microfluidic chip. Following the protocol of the GEXSCOPE Single Cell Immuno‐TCR/BCR Kit, scTCR‐seq/scBCR‐seq libraries were constructed. The mRNAs captured were reverse transcribed into complementary DNA (cDNA) and amplified. Partial cDNA was fragmented and ligated to create a sequencing library suitable for the Illumina sequencing platform. The remaining cDNA was enriched for immune receptors, and the enriched product underwent polymerase chain reaction (PCR) amplification to construct an additional sequencing library for the Illumina platform. Subsequently, the libraries were sequenced on the Illumina Novaseq 6000 platform. Finally, CeleScope v1.5.2 with default parameters was used for scRNA‐seq data alignment and quantification, while Cell Ranger v4.0.0 was employed for TCR clonotype assignment, with GRCh38 used as the reference genome.

### 4.4 Data collection

The expression array data of 20 T‐LBL and 10 T‐ALL samples were retrieved from NCBI Gene Expression Omnibus (GEO) repository (GSE29986^8^). To investigate the impact of MPs, RNA‐seq data from the TARGET‐ALL‐P2 cohort were sourced from The Cancer Genome Atlas (TCGA) database. Four scRNA‐seq datasets were retrieved from publicly accessible repositories to further compare the distinctions between T‐LBL and T‐ALL. Among them, the scRNA‐seq data of thymus cells from healthy thymus samples (T03, T06 and T07) and an epithelial cell sample (T07) were obtained from the European Nucleotide Archive (ENA) database (E‐MTAB‐8581^20^). The scRNA‐seq data of three healthy paediatric BM samples (BMMC‐1, BMMC‐2 and BMMC‐3) and two T‐ALL BM samples (Pre‐TALL1 and Pre‐TALL2) at diagnosis were obtained from the GEO database (GSE132509^19^). Furthermore, the scRNA‐seq data of 10 T‐ALL BM samples (TALL1–TALL10) at diagnosis were retrieved from the GEO database (GSE227122^18^). RNA‐seq and RRBS datasets of Uhrf1‐sufficient and Uhrf1‐deficient T cells were retrieved from the GEO database (GSE143974^28^).

### 4.5 Processing of scRNA‐seq data

The Seurat v4.1.1 R package was predominantly employed for the standard analysis of scRNA‐seq data.^32^ First, four scRNA‐seq datasets were processed separately. Cells were classified as low quality if they possessed fewer than 100–500 features or if the proportion of mitochondrial genes was greater than 15%–25%. The specific filtering criteria are detailed in the Supporting Information, depending on the dataset. Doublets were removed via the R package DoubletFinder.^33^ Following this, 2000 highly variable genes were selected and scaled after normalising the gene expression in the qualified cells. Uniform manifold approximation and projection (UMAP) was utilised for dimensionality reduction, which was subsequently followed by cell clustering. Each cell cluster was annotated based on canonical markers. When additional datasets were integrated, batch effect correction was applied to normal cells, excluding malignant T cells, using the Harmony algorithm.

### 4.6 Processing of scTCR‐seq data

Chains were screened based on full‐length recombinant sequences and a minimum of two unique molecular identifier counts. The filtered contig annotations were consolidated and incorporated into the T‐LBL Seurat object by barcode. Clonotypes were classified as single, small, medium, large or hyperextended based on their abundance, with categories determined according to the following ranges: 0, 1, 5, 20, 100 and 2500.

### 4.7 Detection of malignant cells within the scRNA‐seq data

The T cells in the scT‐LBLs and scT‐ALLs were clustered and analysed according to the expression of T‐cell function genes, respectively. Additionally, the inferCNV package was utilised to assess chromosomal CNVs in single cells.^34^ The CNV scores were then combined with the clustering results to identify malignant cells.

### 4.8 Transcriptome similarity evaluation

To determine the overall similarity of malignant T cells in T‐LBL and T‐ALL, we merged all the malignant T cells. After dimensionality reduction, the cell embeddings of the top 30 PCs were extracted, and the mean value of each PC was calculated to represent the transcriptomic features of each sample. The average of the 30 PCs for each sample was subsequently used to calculate Pearson correlation coefficients and evaluate sample distances. Thus, the similarity of these samples was defined as the distance and clustered. The biological function of the first three PCs was defined by the enrichment results of the top 100 genes in terms of absolute value.

### 4.9 Construction of T‐cell differentiation stage classifier by scRNA‐seq data

The scRNA‐seq data sourced from healthy thymus and paediatric BM samples were utilised to construct a T‐cell differentiation stage classifier. We selected CLP/MPP cells from the BM, as well as DN (early), γδT, DN (P), DN (Q), DP (P), DP (Q), αβT (entry), and CD4^+^T and CD8^+^T cells from the thymus. The top 100 feature genes were detected in each cell type via the ‘FindAllMarkers’ function of Seurat. Among them, 854 unique feature genes were used to build the classifier. The detailed classifier construction method was based on the work of Malta et al.^35^ Cells from healthy thymus and BM were divided into training and testing datasets at an 8:2 ratio.

### 4.10 T‐cell differentiation related meta‐program identification

Non‐negative matrix factorisation (NMF) was utilised to T cells from scT‐LBLs and scT‐ALLs to identify gene expression programs.^36^ Hierarchical clustering was then used to determine shared active MPs. The function of each MP was characterised by the top 50 scoring genes from the shared gene programs. The activity of each MP in individual cells was calculated via the ‘AddModuleScore’ function in Seurat. The similarity between MPs and the differentiation stages of T cells was assessed by computing the Pearson correlation coefficient between the MP activity of the samples and the proportion of T cells at each differentiation stage. For the RNA‐seq data, the MP activity was calculated by GSVA.^37^

### 4.11 Methylation sequencing and analysis

After DNA extraction, WGBS libraries were fabricated by the Acegen Bisulfite‐Seq Library Prep Kit (AG0311, Acegen). The eligible libraries were then sequenced on an Illumina NovaSeq X Plus. After quality screening, the reads were aligned to the GRCh38, and the methylation levels at individual sites were calculated via BSMAP v 2.7.3. Next, metilene^38^ was applied to analyse the DMRs for the WGBS data, and the R package DSS was utilised to identify DMLs for the RRBS data from GSE143974. For DMRs, the default filter criterion of metilene was used; for DMLs, the thresholds were set at delta = .15 and *p*.threshold = .001.

### 4.12 RNA sequencing and analysis

After RNA extraction, total RNA libraries were generated using the Acegen RNA Library Prep Kit (AG2402, Acegen). Qualified libraries were subsequently sequenced on the Illumina NovaSeq X Plus platform. Following quality assessment, the reads were aligned to the GRCh38, and featureCounts was employed to quantify gene expression levels.

### 4.13 Differentially expressed genes and functional enrichment

DEGs from the microarray gene expression data obtained from GSE29986 were analysed using the R package limma, with thresholds set at |log_2_ fold change| >1 and adjusted *p* value <.05. For RNA‐seq data, DEGs were calculated using the DESeq2 by the Wald test. Regarding the RNA‐seq data of shUHRF1 knockdown, the thresholds were set at |log_2_ fold change| >.5 and adjusted *p* value <.05, and for the RNA‐seq data of GSE143974, the thresholds were set at |log_2_ fold change| >.2 and *p* value <.01. For the scRNA‐seq data, DEGs were detected using the ‘FindMarkers’ function of the Seurat package, with thresholds set at |avg log_2_ fold change| >.25 and adjusted *p* value <.01. For transcription factors, the activities of transcription factors in T cells at different differentiation stages were calculated using the Python package pySCENIC.^39^ These data were then analysed with the ‘FindMarkers’ function from Seurat to identify active transcription factors in the DN and DP stages. GSEA was executed with the clusterProfiler package,^40^ and the hallmark gene set for apoptosis was downloaded from Molecular Signature Database.

### 4.14 Cell communication pathways inference

Cell–cell communication pathways in the thymus and T‐LBL were inferred using CellChat version 1.5.0,^39^ with the *CellChatDB human* database selected as the reference for cell communication analysis. The interaction strengths among the cells were visualised using a chord diagram. The role of the MHC pathway was assessed at each differentiation stage of T cells.

### 4.15 Cell culture, drug treatment, and cell viability

SUP‐T1 (DP cells from childhood T‐lymphoblastic lymphoma^26^) and Jurkat (single CD4^+^ T cells from T‐ALL^41^) cells were cultured in RPMI 1640 medium fortified with 10% foetal bovine serum. All the cells were maintained in an incubator at 37°C with 5% carbon dioxide. For the drug efficacy assay, cells were plated in 96‐well plates at an appropriate density and treated with a series of azacitidine concentration gradients for 72 h. Then cell viability was determined by the CCK‐8 assay (k1018, Apex Bio), with the absorbance measured 3 h after the addition of CCK‐8.

### 4.16 Knockdown of UHRF1 in cell lines

A lentivirus with green fluorescent protein (GFP) fluorescence was used to knockdown UHRF1 in SUP‐T1 cells via shRNA interference. The cells were then selected using 2 µg/mL of puromycin. The sequences used are as follows: shRNA‐1 (GCGAGAGAAGGAGAACAGCAA), shRNA‐2 (GCTGGAGGAGGACGTCATTTA) and vector (TTCTCCGAACGTGTCACGT).

### 4.17 Quantitative real‐time PCR

RNA was extracted from cells with TRIzol (15596026CN, Thermo Fisher Scientific) and reverse‐transcribed into cDNA with 5×HiScript II QRT SuperMix (R223‐01, Vazyme). Then, qRT‐PCR was conducted on a Roche LightCycler 480 with the fluorescent dye SYBR Green I Master. ACTB served as the reference gene for normalising the transcriptional abundances of target genes.

### 4.18 Western blotting

The protein concentration was quantified using the bicinchoninic acid (BCA) assay (P0012, Beyotime) and then processed according to the standard Western blotting (WB) protocol. The following primary antibodies were employed for incubation: UHRF1 (1:2000, ab213223, Abcam), β‐actin (1:5000, 30102es40, YEASEN), β‐Tubulin (1:2000, A12289, ABclonal), FOXP1 (1:2000, A23442, ABclonal), PIK3R1 (1:2000, 60225‐1‐Ig, Proteintech) and KLF6 (1:2000, A10011, ABclonal). After incubation with the primary antibodies, horseradish peroxidase (HRP)‐labelled secondary antibodies (1:5000, SA00001‐1, SA00001‐2, Proteintech) were applied. Chemiluminescence substrates were used for protein imaging on a Bio‐Rad ChemiDoc XRS+, and densitometric analysis was performed via ImageJ software.

### 4.19 Apoptosis detection by Annexin V and PI

Given that the cells displayed GFP fluorescence, the apoptosis levels of UHRF1 knockdown and vector control cells were evaluated using an Annexin V‐APC/PI Apoptosis Detection Kit (A214‐01, Vazyme). For SUP‐T1 cells treated with 4 µM azacitidine (HY‐10586, MedChemExpress) for 72 h, their apoptosis was determined with an Annexin V‐FITC/PI Apoptosis Detection Kit (A211‐01, Vazyme). Following staining, the cells were promptly analysed by flow cytometry.

### 4.20 Cell cycle assessment

A cell cycle analysis kit (C1052, Beyotime) was used to assess the cell cycle distribution of the vector control, UHRF1‐knockdown and SUP‐T1 cells treated with 4 µM azacitidine for 72 h. After harvesting, the cells were fixed using 70% ethanol and stained. Subsequently, flow cytometry analysis was performed to assess the cell cycle distribution.

### 4.21 Dual‐luciferase reporter assay

A total of 5 × 10⁵ HEK293T cells were seeded into 12‐well plates. Once they reached 60% confluence, the cells were transfected for 24 h with pGL3‐Basic‐mUHRF1 promoter constructs (NJ0142633‐1, Beijing Tsingke Biotechnology) and E2F2 plasmids (PPL01501‐4a, Public Protein/Plasmid Library). Following transfection, the HEK‐293T cells were collected. The Dual Luciferase Reporter Gene Assay Kit (E1910, Promega) was then employed to measure the activity of both firefly and Renilla luciferases via a Promega luminometer. The firefly luciferase activity was normalised relative to the corresponding Renilla luciferase activity.

### 4.22 Chip‐qPCR

The E2F2–DNA complex was obtained using the Simple ChIP Plus Enzymatic Chromatin IP Kit (#9002, Cell Signaling Technology) with an E2F2 antibody (sc‐514477 X, Santa Cruz Biotechnology). The complex was then subjected to qPCR using primers containing the E2F2/UHRF1 binding motif, which is UHRF1 (F): GGTGAGCCGAGATTGCGCCA and UHRF1 (R): AGAGAGGCTGGGGCTGCAGT. The results were subsequently calculated as the fold difference in the target DNA region enriched by the E2F2 antibody compared with that enriched with the control IgG antibody.

### 4.23 Immunohistochemistry and immunofluorescence

The slides of mediastinal punctures from T‐LBL patients, normal human lymph nodes and tumours from mice were processed by fixation with 10% formalin, followed by paraffin embedding, and sectioned into 4 µm slices. For human pathology slides, one part was stained for UHRF1 according to the immunohistochemistry protocol via an anti‐UHRF1 antibody (1:100, Abcam, ab213223), and the other part was subjected to protein detection via multicolour immunofluorescence via the following antibodies: CD4 (1:2000, GB13588, Servicebio), CD8 (1:2000, GB12068, Servicebio) and PP2Ac (1:2000, 13482‐1‐AP, Proteintech) or CD4, CD8 and LGALS9 (1:2000, 17938‐1‐AP, Proteintech). For mouse tumour slides, we examined changes in target protein expression following demethylation treatment with the following antibodies: FOXP1 (1:200, A23442, ABclonal), PIK3R1 (1:200, 60225‐1‐Ig, Proteintech) and KLF6 (1:200, A10011, ABclonal). Subsequently, Image‐Pro Plus software was employed to quantify relative positive areas of the target proteins, with each slide analysed across five fields of view.

### 4.24 Statistical analysis

Data analysis was conducted using statistical tests appropriate to the data type. Specifically, Student's *t*‐test, Fisher's exact test or one‐way ANOVA with Holm–Sidak's multiple‐comparison test were applied. These analyses were carried out with SPSS (version 21.0) and R software (4.2.1), statistical significance was set at a *p* < .05. For visualisation, R packages such as ggplot2, pheatmap, ComplexHeatmap and ggvenn were utilised. Additionally, other packages employed in each analytical step have been previously described.

## AUTHOR CONTRIBUTIONS

Yi‐Xuan Zhang, Jiali Wang, Bo Qian, Yaping Wang, Yongjun Fang and Gang Zhang designed the research; Jiali Wang and Yi‐Xuan Zhang conducted the analysis and visualisation of the sequencing data and cohort data; Jiali Wang, Xiaowen Yu, Bo Qian, Yidan Zhang, Tingting Yang, Le Xia and Chunlei Zhou performed the experiments; Yi‐Xuan Zhang and Jiali Wang wrote the paper; Yi‐Xuan Zhang, Yaping Wang and Yongjun Fang supervised the overall research, secured funding and interpreted the results.

## CONFLICT OF INTEREST STATEMENT

The authors declare no conflicts of interest.

## ETHICS STATEMENT

This study was approved by the Ethics Committee of Children's Hospital of Nanjing Medical University (Ethics No. 202307011‐2, 201902046‐1, 202008043‐1) and was conducted in accordance with the Declaration of Helsinki. Informed consent was obtained from all patients or their parents. Animal care and experimental procedures were sanctioned by the Animal Welfare Ethics Committee of Nanjing Medical University (Ethics No. IACUC‐2410024) in compliance with Chinese animal welfare laws.

## Supporting information



Supporting Information

Supporting Information

Supporting Information

Supporting Information

Supporting Information

Supporting Information

Supporting Information

Supporting Information

Supporting Information

Supporting Information

Supporting Information

Supporting Information

Supporting Information

Supporting Information

Supporting Information

Supporting Information

Supporting Information

## Data Availability

The scRNA‐seq and scTCR data of the T‐LBL patients used in this study can be obtained from the Science Data Bank (accession number: 31253.11. scientificdb. 15166) through dataset link (https://cstr.cn/31253.11.sciencedb.15166). The remaining data used to support this study are stored in the Supporting Information Table.
