# Supplementary material for: T‐cell differentiation stage block bias confers hypermethylation and mediastinal preference in T‐cell lymphoblastic lymphoma
Source: Clin Transl Med. 2025 Jun 27;15(7):e70380. doi: 10.1002/ctm2.70380 (PMC12205001; doi:10.1002/ctm2.70380)
Supplement: Supplementary file 17 — Supporting Information [file CTM2-15-e70380-s004.docx]

Supplemental Figure 1

**
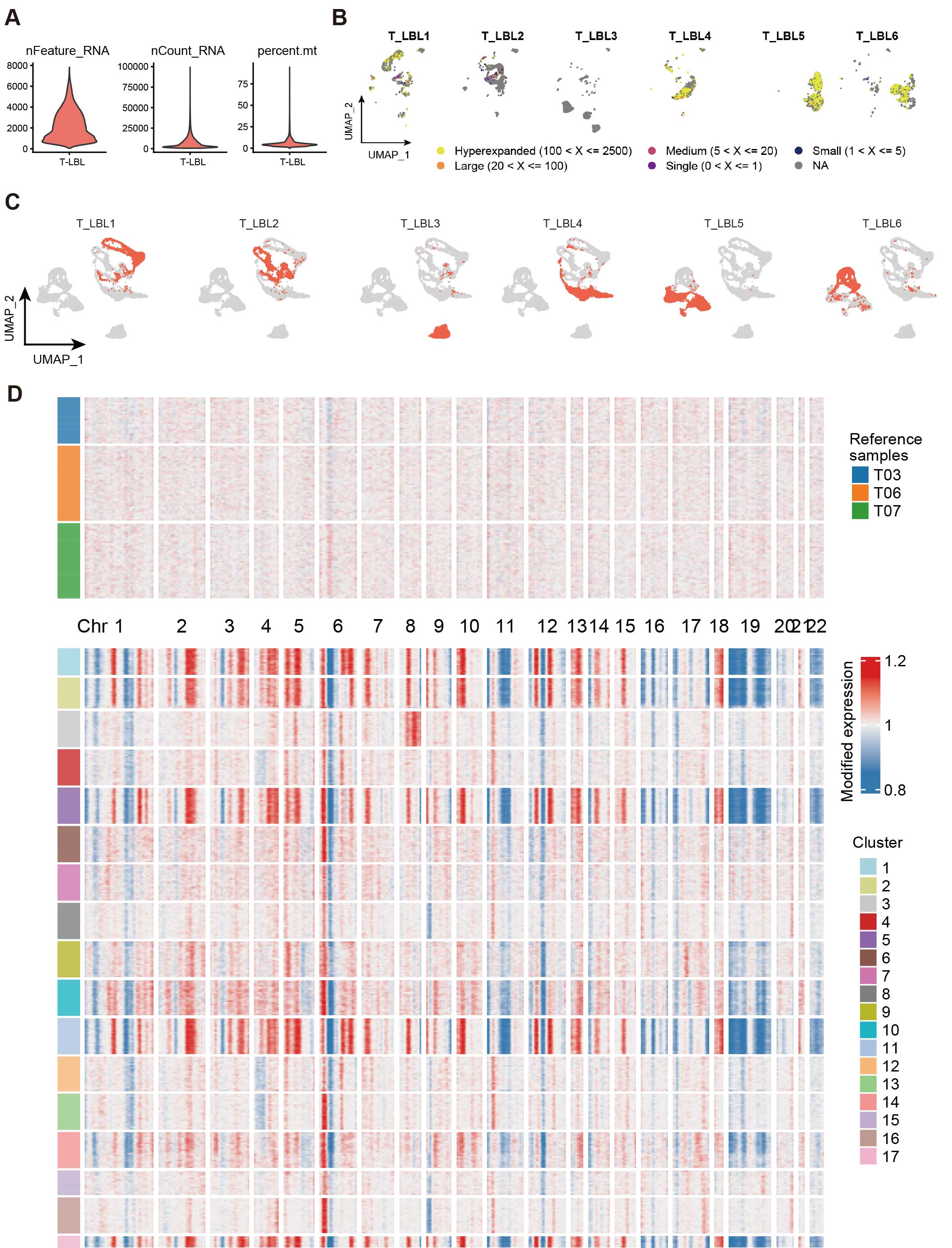
**

A. Quality control metrics for scT-LBL, with filtering criteria set as follows: nFeature_RNA> 500 and < 6,000, percent.mt < 20, nCount_RNA < 40,000.

B. TCR frequency distribution in each sample of scT-LBLs.

C. Distribution of T cell clusters for each T-LBL patient in UMAP.

D. Copy number variations (CNVs) in each T cell clusters of scT-LBL, calculated using InferCNV. The reference normal thymus cells were from thymus samples (T03, T06, T07).

Supplemental Figure 2


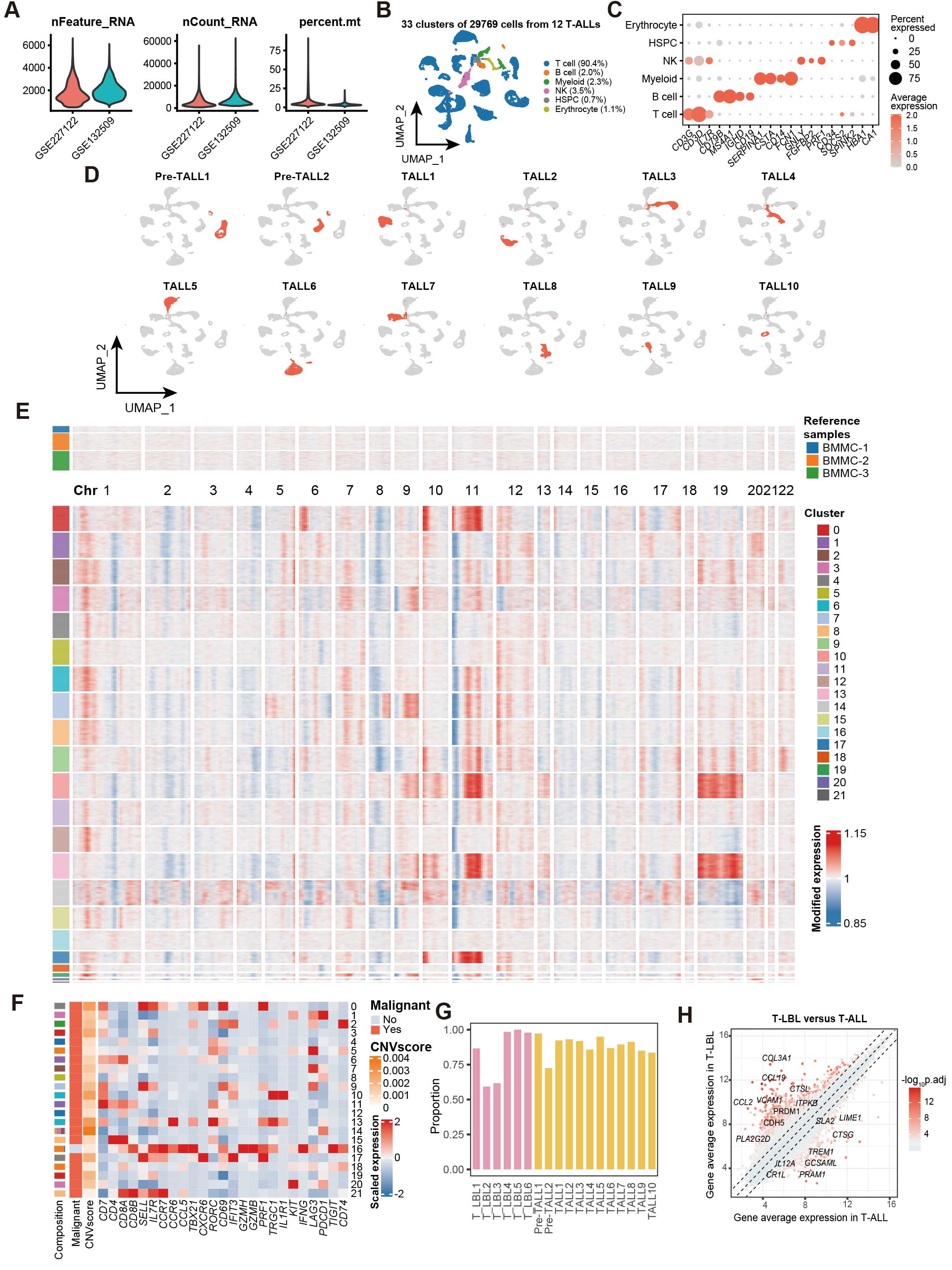


A. Quality control metrics for scT-ALL, with filtering criteria set as follows: nFeature_RNA> 100 and < 6,000, percent.mt < 25, nCount_RNA < 40,000.

B. Two-dimensional visualization of annotated major cell types from scT-ALLs by UMAP.

**C.** Dotplot illustrating the average expression of canonical markers in each cell type of scT-ALLs.

**D.** Distribution of T cell clusters for each T-ALL patient in UMAP.

**E.** CNVs in each T cell clusters of scT-ALL, calculated using InferCNV. The reference normal T cells were from bone marrow samples (BMMC-1, BMMC-2, BMMC-3).

**F.** Heatmap indicating the expression levels of T cell function genes in each T cell cluster of scT-ALLs, with patient compositions, malignant status and CNV scores displayed in annotated columns.

**G.** Proportion of malignant T cells in scT-LBLs and scT-ALLs.

**H.** DEGs in T-LBL versus T-ALL from GSE29986; red dots beyond the dotted lines represent DEGs, with the dotted line indicating the average gene expression with a twofold change threshold.

Supplemental Figure 3

**
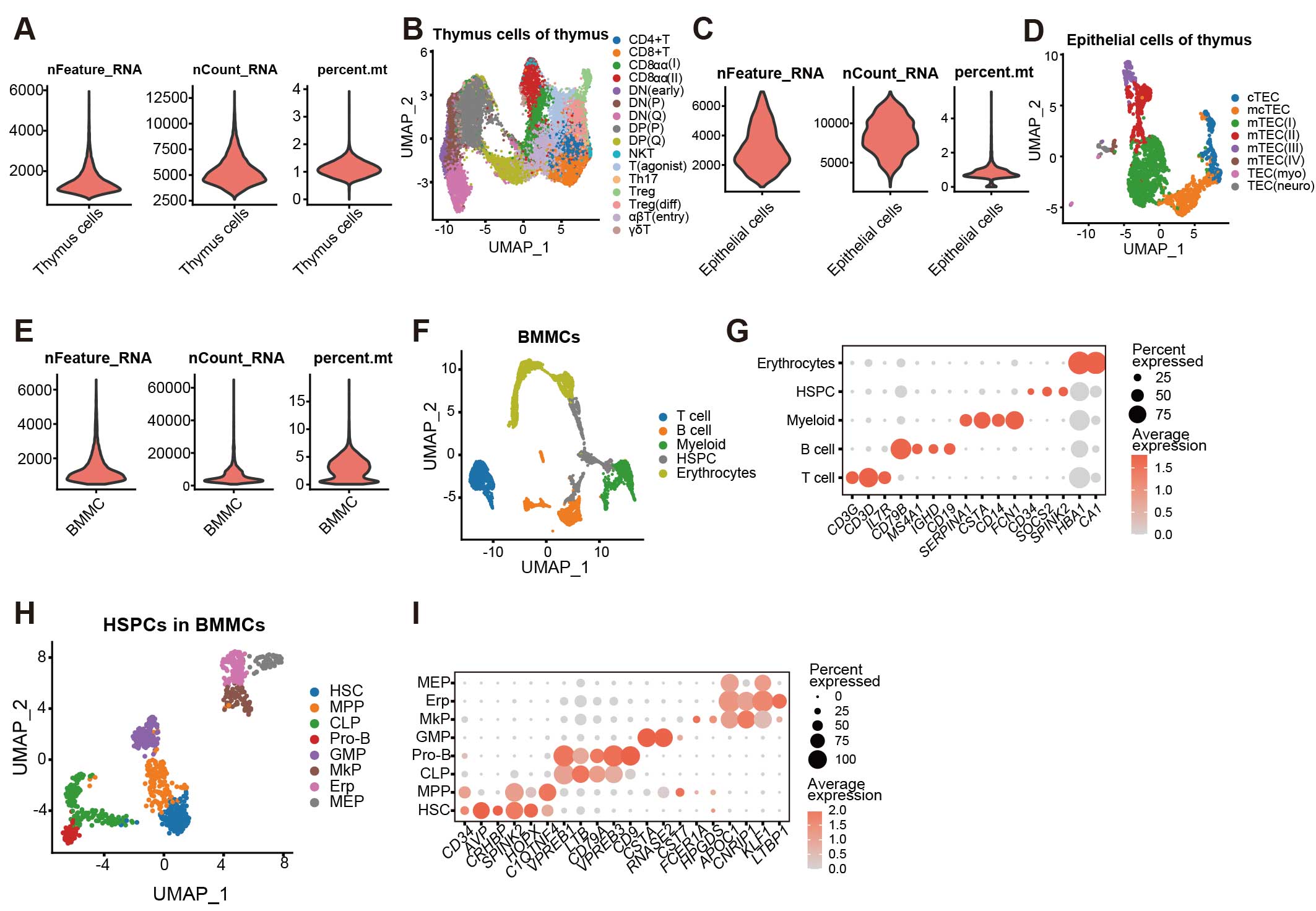
**

A. Quality assessment of scRNA-seq data from normal thymus cells derived from three healthy thymus samples (T03, T06, T07).

B. Two-dimensional visualization of thymus cells at each differentiation stage from three healthy thymus samples (T03, T06, T07) by UMAP.

C. Quality assessment of scRNA-seq data from epithelial cells derived from a healthy thymus sample (T07).

D. Two-dimensional visualization of different types of epithelial cells from a healthy thymus sample (T07) by UMAP.

E. Quality control metrics for scRNA-seq from BMMCs, with filtering criteria set as follows: nFeature_RNA > 100 and < 6000, percent.mt < 15, nCount_RNA < 60000.

F. Two-dimensional visualization of annotated major cell types from scRNA-seq of BMMCs by UMAP.

G. Dotplot representing the average expression of canonical markers in each cell type of scRNA-seq of BMMCs.

H. Two-dimensional visualization of the fine cell types of HSPCs from scRNA-seq of BMMCs by UMAP.

I. Dotplot representing the average expression of canonical markers in each cell type of HSPCs.

Supplemental Figure 4

**
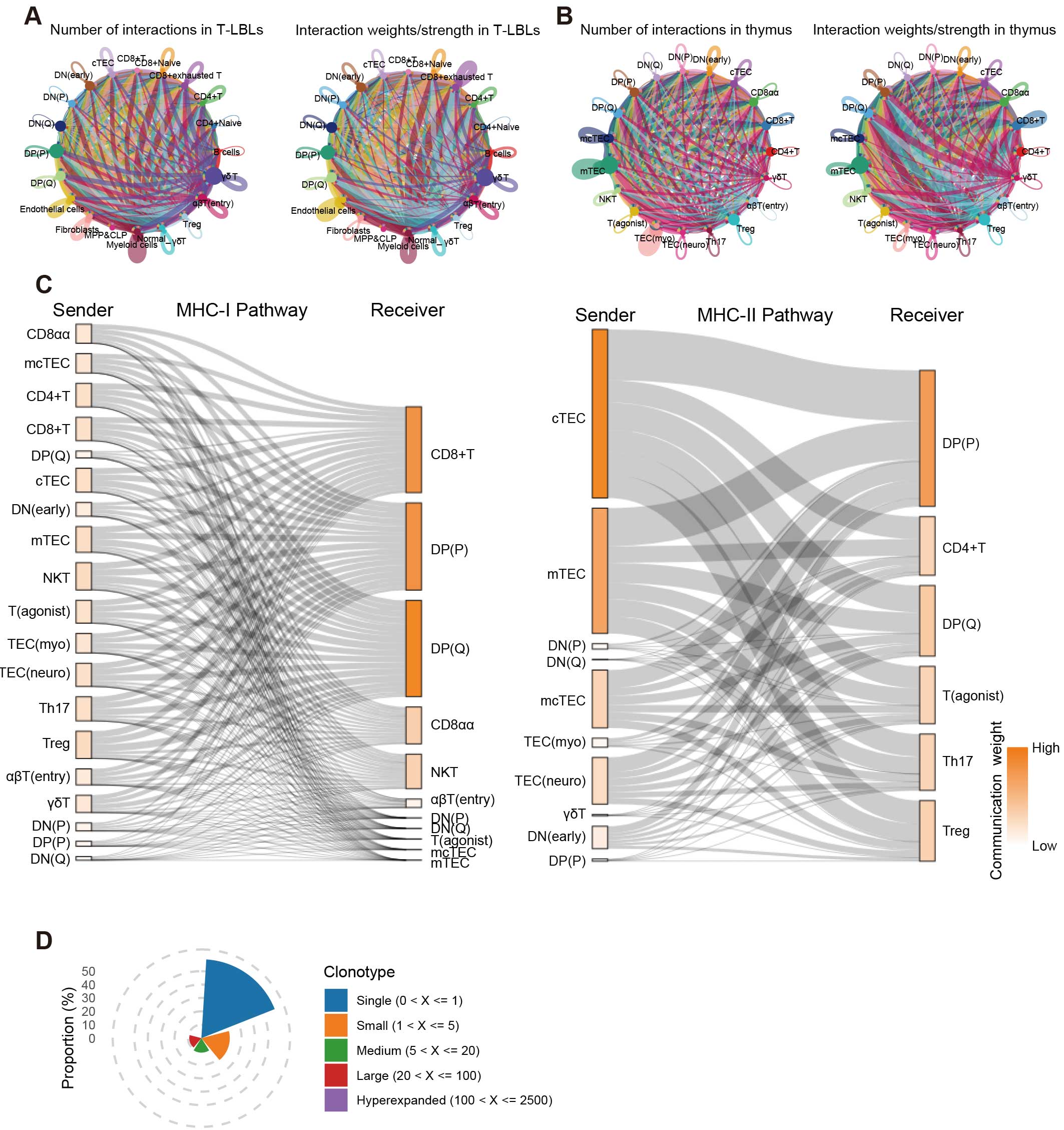
**

A. Cell communication relationship in T-LBLs.

B. Cell communication relationship of thymus cells and epithelial cells from a healthy thymus sample (T07).

C. Sankey diagram illustrating the communication weights of MHC-I (left) and MHC-II (right) between source and target cells in healthy thymus sample (T07).

D. Distribution of TCR clone frequencies of Normal T cells in T-LBL.

Supplemental Figure 5

**
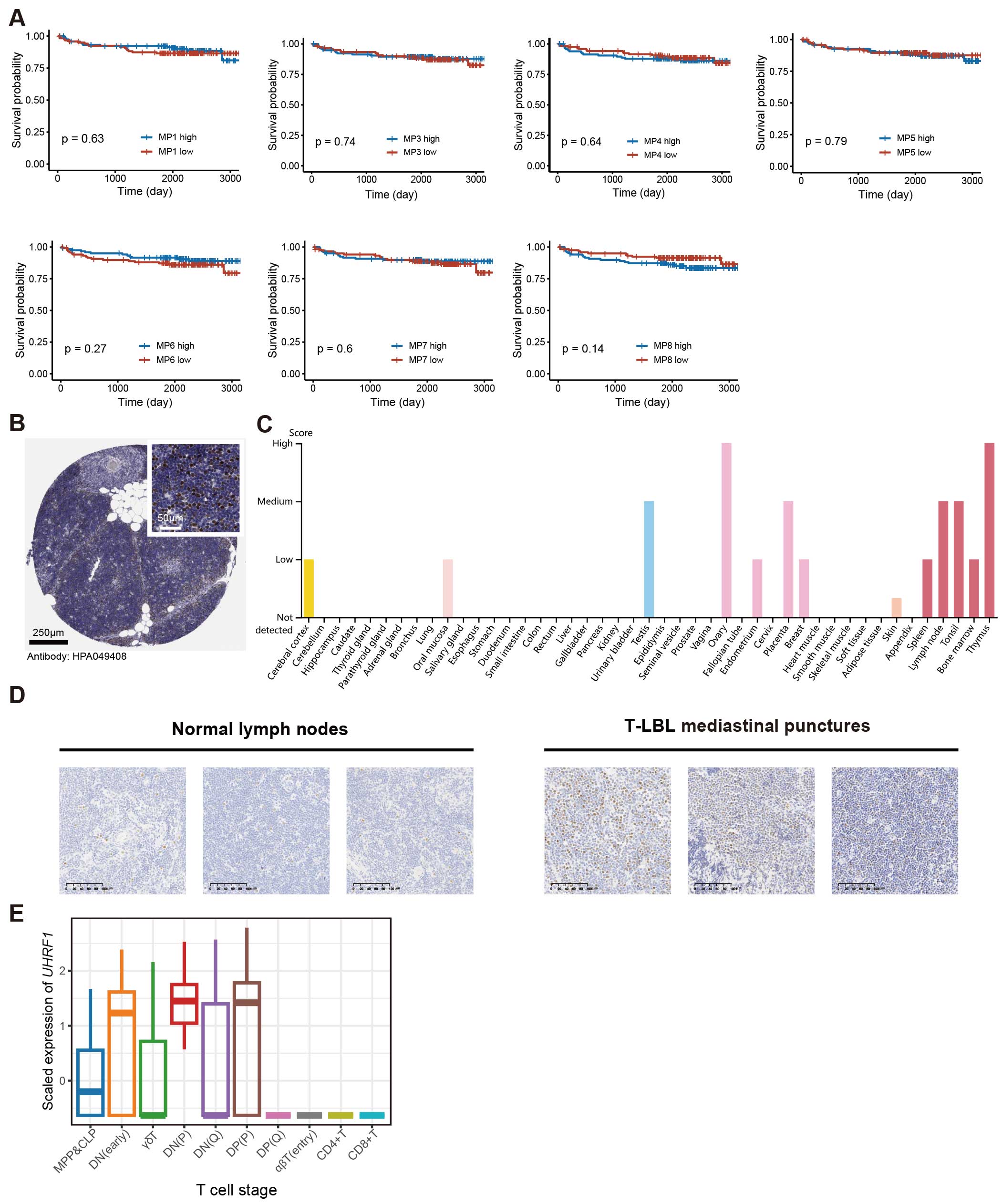
**

A. Kaplan-Meier curves of other seven MPs, p-values were calculated by log-rank test.

B. Immunohistochemistry of UHRF1 in thymus from the Human Protein Atlas database.

C. Protein abundance of UHRF1 across various organs from Human Protein Atlas database.

D. Immunohistochemistry of UHRF1 in in mediastinal punctures of T-LBLs and normal lymph nodes (n=3).

E. Boxplot demonstrating that UHRF1 is highly expressed in the early differentiation stages of thymus cells, based on the scRNA-seq data from healthy thymus samples.

Supplemental Figure 6

**
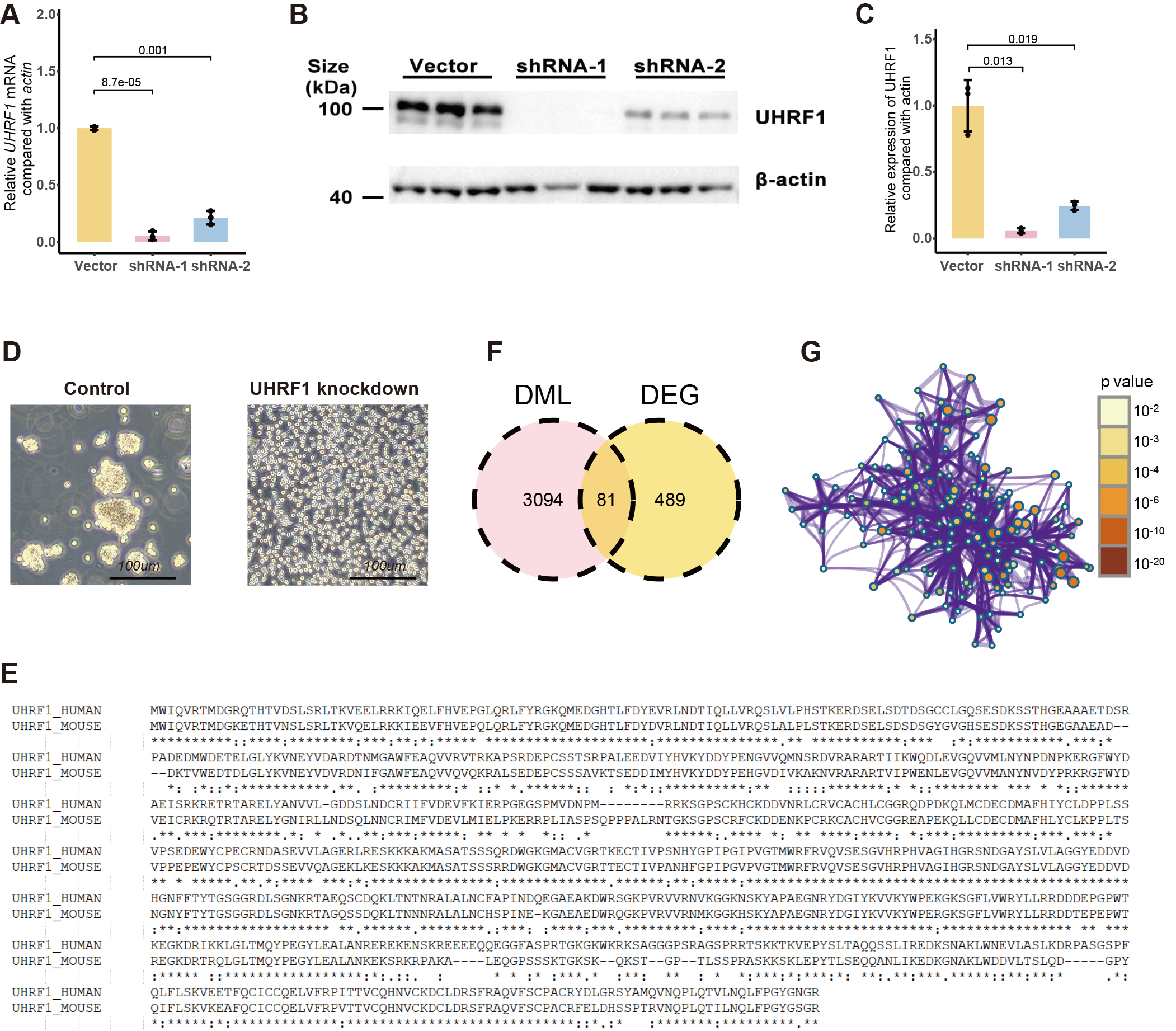
**

A. Barplot showing the knockdown efficacy of UHRF1 in SUP-T1 as assessed by qPCR.

B. WB demonstrating the knockdown efficacy of UHRF1 in SUP-T1.

C. Bar plot indicating that shRNA-1 exhibits the highest knockdown efficiency as determined by WB, and is therefore selected for subsequent experiments.

D. Morphological changes observed in UHRF1 knockdown cells.

E. Conservation of the amino acid sequence of UHRF1 between humans and mice, analyzed using Clustal Omega. Asterisks (*) or colons (:) denote conserved amino acid residues.

F. Intersection of DMLs and DEGs of GSE143974.

G. P values of each enriched item of Metascape network related to Figure 6C.

Supplemental Figure 7

**
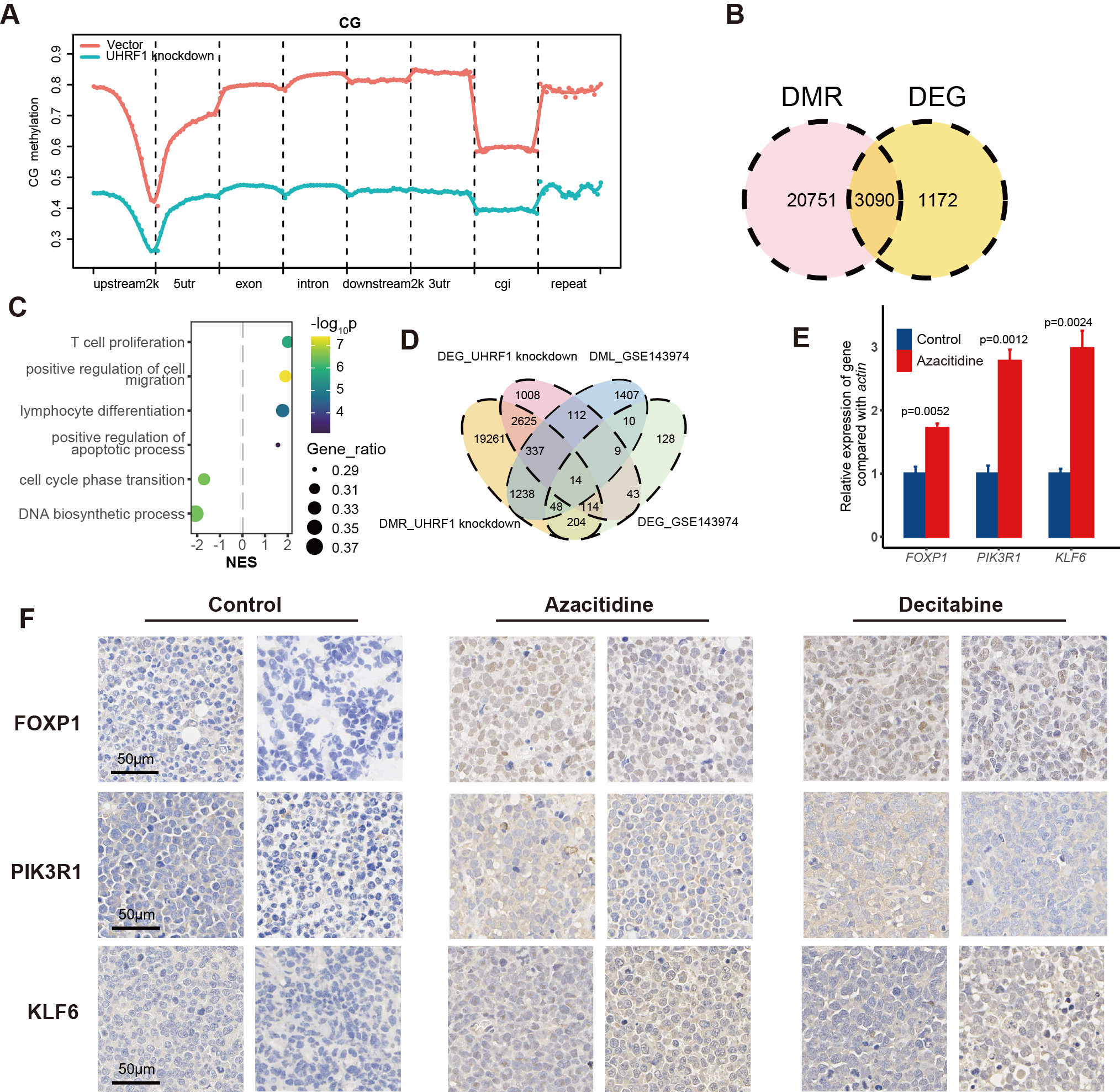
**

A. Comparison of CG methylation levels in functional elements of the genome between vector control and UHRF1 knockdown cells.

B. Intersection of DMRs and DEGs of vector control and UHRF1 knockdown cells.

C. The different pathways enriched by GSEA between vector control and UHRF1 knockdown cells.

D. Final intersected genes of GSE143974 and UHRF1 knockdown cells.

E. Barplot showing the up-regulated of target genes after SUP-T1 treated with azacitidine through qPCR. p values were calculated by two-tailed t-test.

F. Remaining immunohistochemistry results related to Figure 7G.
